# Evaluation of the phytochemical profile, biological activities, and acute toxicity of *Cistus ladanifer* aerial part extracts: experimental and *in silico* investigations

**DOI:** 10.3389/fchem.2026.1911960

**Published:** 2026-09-22

**Authors:** Fatima Zahra Ayyad, Mouad Lahyaoui, Hajar Lahyaoui, Morad Kaddouri, Mohamed El Yaqoubi, Mouhcine Hayani, Amina Bari, Ahde El Imache, Joe Miantezila Basilua, Abdelaaty A. Shahat, Mohammed S. Alharbi, Touriya Zair

**Affiliations:** 1 Research Team of Chemistry of Bioactive Molecules and the Environment, Laboratory of Innovative Materials and Biotechnology of Natural Resources, Faculty of Sciences, Moulay Ismaïl University, Meknes, Morocco; 2 Laboratory of Innovative Technologies, Process Engineering Department, Higher School of Technology Fez, Sidi Mohamed Ben Abdellah University, Fez, Morocco; 3 Laboratory of Applied Organic Chemistry, Faculty of Science and Technology, Sidi Mohamed Ben Abdellah University, USMBA, Fez, Morocco; 4 Laboratory of the Engineering and Applied Technologies, Higher School of Technology, Sultan Moulay Slimane University, Beni Mellal, Morocco; 5 Laboratory of Biotechnology, Environment, Agri-food, and Health, Faculty of Sciences, Sidi Mohamed Ben Abdellah University, Fez, Morocco; 6 Department of Biostatistics and Mathematics, Faculty of Pharmacy, Paris Cité University, Paris, France; 7 Department of Pharmacognosy, College of Pharmacy, King Saud University, Riyadh, Saudi Arabia; 8 Center for Research on Medicinal, Aromatic, and Poisonous Plants, DSR, King Saud University, Riyadh, Saudi Arabia

**Keywords:** antihyperglycemic, antimicrobial, antioxidant, *Cistus ladanifer*, *in silico*, toxicity

## Abstract

**Introduction:**

Using both experimental and *in silico* approaches, this study aimed to evaluate the phytochemical composition and biological activities of *Cistus* *ladanifer* aerial part extracts.

**Methods:**

Cistus ladanifer aerial parts were collected in the Khenifra region, Morocco. Hydroethanolic (Soxhlet) and aqueous (decoction) extracts were prepared. Physicochemical parameters, mineral content (ICP-AES), phytochemical screening, total polyphenols, flavonoids, and condensed tannins were determined. HPLC/UV-ESI-MS was used for chemical profiling. Antioxidant activity was assessed by TAC, DPPH, and FRAP assays; antimicrobial activity by MIC and MBC/MFC against bacterial and fungal strains; acute toxicity in mice at 2 g/kg; antihyperglycemic activity by oral glucose tolerance test in rats; and molecular docking against selected antioxidant, antimicrobial, and antihyperglycemic protein targets.

**Results:**

Physicochemical analysis of the plant material showed a moisture content of 12.68% ± 0.42%, pH of 4.16 ± 0.05, and ash content of 5.50% ± 0.31%. Mineral analysis revealed essential elements such as Zn, Mn, Fe, and Al. Phytochemical screening confirmed major secondary metabolites, including flavonoids, tannins, saponins, and alkaloids. The hydroethanolic extract exhibited the highest extraction yield (23.81% ± 0.63%), whereas the aqueous extract (*CL2*) was richer in total polyphenols (433.89 ± 1.82 mg GAE/g DE), flavonoids (54.13 ± 0.52 mg CE/g DE), and condensed tannins (19.50 ± 0.87 mg CE/g DE). HPLC/UV-ESI-MS analysis revealed the presence of several phenolic compounds, including resveratrol 4′-(6-galloylglucoside), kaempferol, and quercetin 3.4′-diglucoside. *CL2* exhibited strong antioxidant activity with a total antioxidant capacity of 462.04 ± 0.41 mg EAA/g DE, a DPPH IC50 of 7.06 ± 0.31 μg/mL, and a FRAP EC50 of 10.42 ± 0.83 μg/mL. Antimicrobial assays showed notable inhibitory effects against several microorganisms, particularly *Candida* *kefyr* and *Saccharomyces cerevisiae* (MIC = 4.69 mg/mL). In addition, *CL2* demonstrated significant antihyperglycemic activity *in vivo* and showed no observable signs of toxicity or mortality at an oral dose of 2 g/kg. Molecular docking analysis further reinforced these results by showing favorable interactions of major phytochemicals with target proteins associated with the investigated biological activities.

**Discussion:**

Our findings highlight *C. ladanifer* as a potent source of bioactive compounds with promising therapeutic applications.

## 1 Introduction

Medicinal plants remain a major source of therapeutic agents and bioactive molecules worldwide (El Hachlafi et al., 2024). Natural products continue to offer a rich reservoir of structurally diverse compounds with potential applications in the prevention and management of several diseases, although their systematic characterization and development remain challenging (Atanasov et al., 2021; Medina-Franco, 2021). The continued use of medicinal plants in traditional healthcare further underlines the importance of investigating their chemical composition and biological properties (Smit et al., 2012).

The genus *Cistus* L (family Cistaceae) is widely distributed throughout the Mediterranean basin and is well adapted to the characteristic environmental conditions of Mediterranean ecosystems, including summer drought and recurrent disturbances (Comandini et al., 2006; Tomou et al., 2022). *Cistus* species are recognized as important sources of secondary metabolites and have attracted considerable interest because of their biological, aromatic, and pharmacological properties (Papaefthimiou et al., 2014). Among them, *Cistus ladanifer* is a perennial Mediterranean shrub adapted to warm, dry, and acidic habitats (Teixido and Valladares, 2015). Its production of labdanum has contributed to its importance in the fragrance and perfumery industries, while recent studies have highlighted the broader valorization potential of its essential oil, extracts, resin, and by-products (Frazão et al., 2018; Gago et al., 2025).

The richness of *C. ladanifer* in secondary metabolites has stimulated increasing scientific interest in its biological properties and potential applications. Recent studies have underscored its value as a source of bioactive compounds with promising pharmaceutical, cosmetic, and food-related applications (Bouothmany et al., 2024; Frazão et al., 2024; Gago et al., 2025). In particular, labdanum resin has been investigated as a natural and sustainable ingredient for skincare formulations because of its antioxidant, anti-inflammatory, antimicrobial, anti-elastase, and photoprotective properties (Frazão et al., 2022). Its essential oil has also attracted attention because of its antioxidant and antibacterial activities, supporting its potential use as a natural bioactive ingredient (Benali et al., 2024).

In Moroccan traditional medicine, *C. ladanifer* has been used for the management of dermatological, gastrointestinal, respiratory, neurological, metabolic, and inflammatory disorders (El Hachlafi et al., 2024; Frazão et al., 2024). Its essential oil and extracts have demonstrated antioxidant, antimicrobial, anti-inflammatory, hypoglycemic, hepatoprotective, and antiproliferative properties (Benali et al., 2024; Blasco et al., 2024; Bouothmany et al., 2024; Chaloupková et al., 2024; El Hachlafi et al., 2024). Among the microorganisms investigated in antimicrobial studies, *Staphylococcus aureus*, *Escherichia coli*, *Klebsiella pneumoniae*, and *Pseudomonas aeruginosa* are clinically important pathogens associated with various human infections (Nortjie et al., 2022). These biological activities have been associated with diverse secondary metabolites, particularly flavonoids, phenolic acids, tannins, and other phenolic constituents (Bouothmany et al., 2024).

The biological activities investigated in the present study are closely related to the phytochemical composition of the aerial parts. Phenolic and other secondary metabolites may contribute to antioxidant activity by scavenging reactive species and limiting oxidative damage, while the same classes of compounds may exert antimicrobial effects through interactions with microbial membranes, enzymes, and other cellular targets (Benam et al., 2024; Muscolo et al., 2024). Antioxidant and phenolic-rich extracts are also of interest in the context of metabolic disorders, particularly diabetes mellitus, in which oxidative stress and chronic hyperglycemia are closely associated with disease progression and complications (Hussain, 2025; Muscolo et al., 2024). Consequently, evaluating antioxidant, antimicrobial, and antihyperglycemic activities within the same phytochemical framework may help offer a more comprehensive understanding of the biological potential of *C. ladanifer* aerial part extracts. However, biological efficacy alone is insufficient to support their potential valorization; assessment of acute toxicity is also necessary to give preliminary information on their safety.

Despite the recognized biological and valorization potential of *C. ladanifer*, few studies have simultaneously combined the phytochemical characterization of its aerial part extracts with antioxidant, antimicrobial, and antihyperglycemic evaluations, acute toxicity assessment, and *in silico* investigation. In particular, the relationship between the phytochemical composition of the aerial parts and these different biological responses has not been sufficiently explored within a single experimental framework. This represents a scientific gap that limits a comprehensive assessment of these extracts as sources of natural bioactive compounds.

Therefore, the present study aimed to present a comprehensive evaluation of *C. ladanifer* aerial part extracts by (i) characterizing their phytochemical profiles, (ii) assessing their antioxidant and antimicrobial activities *in vitro*, (iii) evaluating their antihyperglycemic effects and acute toxicity *in vivo*, and (iv) investigating the potential interactions of selected compounds with relevant molecular targets through *in silico* approaches. This study provides a comprehensive basis for the scientific valorization of *C. ladanifer* aerial parts by linking chemical composition, biological activities, and preliminary safety assessment.

## 2 Materials and methods

### 2.1 Materials

#### 2.1.1 Plant material

*Cistus ladanifer* (*Cistaceae*), commonly known as rockrose, was collected in April 2024 from the Khenifra region, Morocco. The plant material was taxonomically identified by Professor Amina Bari, botanist at the Biology Department, Faculty of Sciences Dhar El Mahraz (FSDM), Sidi Mohamed Ben Abdellah University (USMBA), Fez, Morocco. A voucher specimen was preserved in the herbarium of the Biology Department and assigned the reference number CCL003424K. The aerial parts were cleaned, shade-dried at ambient temperature until completely dehydrated, ground into a fine powder, and stored in airtight containers in a cool, dark place at room temperature until further analysis.

#### 2.1.2 Microbial strains

The antimicrobial activity of *C. ladanifer* aerial part extracts was assessed using nine bacterial strains and eight fungal strains (Table 1). The bacterial strains were isolated from the Mohamed V Provincial Hospital in Meknes, Morocco, while the fungal strains, including yeasts, were obtained from the Mycotheca of the Parasitology-Mycology Laboratory at the Ibn Sina Hospital Center (ISHC), Rabat, Morocco. All isolates were previously identified using the automated BD Phoenix system for identification and antibiotic susceptibility testing. The microorganisms were stored at −80 °C in 20% glycerol until use. Prior to experimentation, strains were reactivated on Mueller–Hinton agar for bacteria and Sabouraud agar for fungi, followed by subculturing to ensure viability and purity.

**TABLE 1:** List of bacterial and fungal strains evaluated.

Bacterial strains	Abbreviations	Fungal strains	Abbreviations
*Staphylococcus aureus* BLACT	*S. aureus* BLACT	*Candida albicans*	*C. albicans*
*Staphylococcus epidermidis*	*S. epidermidis*	*Candida kefyr*	*C. kefyr*
*Streptococcus agalactiae* (B)	*S. agalactiae* (B)	*Candida krusei*	*C. krusei*
*Escherichia coli* ESBL	*E. coli* ESBL	*Candida parapsilosis*	*C. parapsilosis*
*Klebsiella pneumonie*	*K. pneumonie*	*Candida tropicalis*	*C. tropicalis*
*Escherichia coli*	*E. coli*	*Candida dubliniensis*	*C. dubliniensis*
*Proteus mirabilis*	*P. mirabilis*	*Saccharomyces cerevisiae*	*S. cerevisiae*
*Pseudomonas aeruginosa*	*P. aeruginosa*	*Aspergillus niger*	*A. niger*
*Enterobacter cloacae*	*E. cloacae*	​	​

#### 2.1.3 Animals

Acute toxicity study was conducted on albino mice (both sexes, body weight 20–35 g), while the *in vivo* antihyperglycemic activity was evaluated using Wistar rats (both sexes, body weight 200–250 g). All animals were rared under standard laboratory conditions, with a 12 h light/12 h dark phtoperiod, a controlled temperature of 22° C ± 2 °C, and free access to food and water. They were housed in polycarbonate cages (21.5 × 46.5 × 14.5 cm) with wire tops and wood shavings bedding, which was replaced once or twice weekly. All experimental procedures and protocols were performed in accord with ethical guidelines and regulations to ensure animal welfare, with the study being reviewed and approved by the Institutional Animal Care and Use Committee (IACUC) of laboratory Animals at the Faculty of Sciences Dhar El Mehraz, Sidi Mohamed Ben Abdallah University, Fez, Morocco (10/04/2019/LBEAS) (Albus, 2012).

### 2.2 Methods

#### 2.2.1 Quality control of plant material of *Cistus ladanifer*

##### 2.2.1.1 pH determination

This method consisted of combining 2 g of plant material with 10 mL of hot distilled water. The mixture was stirred at room temperature for 5 min, then filtered and allowed to cool. The pH was measured by immersing the electrode of the pH meter in a sufficient volume of the filtrate (Hayani et al., 2022).

##### 2.2.1.2 Moisture content

Moisture content (MC) was calculated in accordance to the AFNOR standard (NF-V03-402, 1985) by comparing the difference in plant mass before and after drying. A 5 g portion of the air-dried plant was placed in Petri dishes and oven-dried at 100° C ± 5 °C for 24 h (NFV03-402, 1985). The Petri dishes were then cooled in a desiccator before reweighing. The moisture content (MC%) was determined using Equation 1:

$$MC\%=\left(\frac{m_{0}-m_{1}}{m_{0}}\right)\times 100$$ (1)

where m_0_: The initial mass of the plant (g); m_1_: The mass of the plant after drying (g).

##### 2.2.1.3 Ash content

The ash content corresponds to the mineral residue remaining after combustion of organic matter (OM) at high temperature in a muffle furnace. Following the AFNOR standard (NF ISO 5984) (NFV05-113, 1972), 2 g of ground plant material was incinerated at 550 °C until all organic components were eliminated, yielding a constant mass of whitish ash. Organic matter content was determined using Equation 2:

$$OM\%=\left(\frac{m_{1}-m_{2}}{TS}\right)\times 100$$ (2)

where m_1_: The mass of the capsule and sample before calcination (g); m_2_: The mass of the capsule and sample after calcination (g); TS: The mass of the test sample (g).

The ash content was calculated using Equation 3:

Ash%=100−OM% (3)

##### 2.2.1.4 Determination of trace metals by ICP-AES

Trace metal concentrations were determined using inductively coupled plasma atomic emission spectrometry (ICP-AES; Ultima 2, Jobin Yvon, HORIBA, Stow, MA, United States of America). Sample preparation followed a standard mineralization protocol (AFNOR, 1999) using aqua regia, enabling the quantification of elements such as, Zn, Co, Mn, Fe, Cu, and Al.

Briefly, 0.1 g of finely ground plant material was mixed with 3 mL of aqua regia, consisting of 1 mL of concentrated nitric acid (HNO_3_; 99%) and 2 mL of hydrochloric acid (HCl; 37%). The mixture was refluxed at 200 °C for 2 h, then cooled and left to settle. The supernatant was collected, filtered through a 0.45 µm membrane, and diluted to 15 mL with distilled water (Skujins, 1998). ICP-AES analysis was performed at the UATRS laboratory (Technical Support Unit for Scientific Research) of the CNRST, Rabat.

#### 2.2.2 Phytochemical screening

Phytochemical screening was conducted to identify primary and secondary metabolites in *C. ladanifer* aerial parts, providing preliminary insight into the chemical profile. The screening involved standard colorimetric and precipitation tests: lipids (plant powder, identified by Liebermann–Burchard test); polysaccharides, proteins, and reducing sugars (5% aqueous decoction, iodine, Biuret/xanthoproteic, and Fehling’s tests, respectively); sterols and triterpenes (10% ether macerate, Liebermann–Burchard test); flavonoids (5% aqueous infusion, cyanidin reaction); tannins (5% aqueous infusion, total tannins by FeCl_3_, catechic/gallic tannins by Stiasny reagent); anthocyanins and leucoanthocyanins (5% aqueous infusion, HCl and isoamyl alcohol tests); saponins (1% aqueous decoction, foam test); monosaccharides and holosides (10% aqueous decoction, Barfoed’s test); alkaloids (10% H_2_SO_4_ macerate, Dragendorff/Wagner reagents); reducing compounds (10% aqueous decoction, Fehling’s test); and mucilages (10% aqueous decoction, alcohol precipitation) (Joshi et al., 2013; Pandey and Tripathi, 2014; Shaikh and Patil, 2020).

#### 2.2.3 Extraction of phenolic compounds

Phenolic compounds were extracted by Soxhlet and decoction methods (Saidi et al., 2023). For the decoction, a mixture of 30 g of plant powder and 600 mL of distilled water was heated at 80 °C for 60 min with stirring, and filtered through filter paper. For Soxhlet extraction, 30 g of plant powder was extracted with 600 mL of ethanol/water mixture (70/30, v/v) using a Soxhlet apparatus until complete extraction. The filtrates from both methods were dried in an oven at 70 °C and then stored as powder in glass vials. Extract codes are presented in Table 2 and extraction yields (Y%) were calculated according to Equation 4:

$$Y\;\left(\%\right)=\left(\frac{m_{2}}{m_{1}}\right)\times 100$$ (4)

where m_2_: Mass of dried extract (g); m_1_: Mass of plant powder (g).

**TABLE 2:** Codes of *Cistus ladanifer* aerial part extracts.

Extract type	Method	Code
Hydroethanolic	Soxhlet	*CL1*
Aqueous	Decoction	*CL2*

#### 2.2.4 Quantification of phenolic compounds

##### 2.2.4.1 Determination of total polyphenol content

Total polyphenol content (TPC) of *C. ladanifer* aerial part extracts was quantified using the Folin-Ciocalteu method as described by Singleton et al. (1999). Briefly, 15 µL of each extract solution (25 mg/mL) was combined with 1.5 mL of 10% (v/v) Folin–Ciocalteu reagent and 1.5 mL of 7.5% (w/v) sodium carbonate solution, diluted to 50 mL with distilled water, and then incubated in the dark at room temperature for 40 min. Absorbance was measured at 760 nm using a UV-Vis spectrophetometer against a reagent blank (reaction mixture without extract). A calibration curve of gallic acid (y = ax + b) was established under identical experimental conditions. TPC was calculated according to Equation 5, and the results were reported as milligrams of gallic acid equivalents per Gram of dry extract (mg GAE/g DE).

$$TPC=\frac{C\times V_{0}}{m_{extract}}\times D$$ (5)

where C: Concentration obtained from the calibration curve (mg/mL); V_0_: Volume of the entire extract (mL); m_extract_: Extract mass (g).

$$D=\frac{V_{f}}{V_{i}}$$

where D: Dilution factor; V_f_: The final volume to be measured in a spectrophotometer (mL); V_i_: The volume taken from the tested extract (mL).

##### 2.2.4.2 Determination of flavonoid content

Flavonoid content (FC) of the extracts was assessed using the aluminum chloride method (Chang et al., 2002). In test tubes, 15 µL of each extract (25 mg/mL) was mixed with 2 mL of distilled water and 10 μL of 10% (m/V) aluminum chloride solution in methanol. The mixture was adjusted to a final volume of 5 mL with pure methanol, thoroughly homogenized, and incubated in the dark at room temperature for 30 min. Absorbance was measured at 433 nm against a reagent blank (reaction mixture without extract). A calibration curve of quercetin (y = ax + b) was established under identical experimental conditions. FC was reported as milligrams of quercetin equivalents per gram of dry extract (mg QE/g DE) and calculated using Equation 6:

$$FC=\frac{C\times V_{0}}{m_{extract}}\times D$$ (6)

where C: Concentration obtained from the calibration curve (mg/mL); V_0_: Volume of the entire extract (mL); m_extract_: Mass of the extract (g).

##### 2.2.4.3 Determination of condensed tannin content

Condensed tannin content (CTC) of *C. ladanifer* aerial part extracts was determined using the vanillin-HCl method (Sun et al., 1998). In test tubes, 30 µL of each extract (25 mg/mL) was combined with 3 mL of 4% (m/v) vanillin solution in methanol and 1.5 mL of 37% HCl. The mixture was thoroughly homogenized and incubated in the dark at room temperature for 20 min. Absorbance was measured at 499 nm against a reagent blank (reaction mixture without extract). A calibration curve of catechin (y = ax + b) was established under identical experimental conditions. CTC was reported as milligrams of catechin equivalents per Gram of dry extract (mg CE/g DE) and calculated according to Equation 7:

$$CTC=\frac{C\times V_{0}}{m_{extract}}\times D$$ (7)

where C: Concentration obtained from the calibration curve (mg/mL); V_0_: Volume of the entire extract (mL); m_extract_: Mass of the extract (g).

#### 2.2.5 HPLC/UV-ESI-MS analysis

Analysis of phenolic composition was performed using high-performance liquid chromatography coupled with UV detection and electrospray ionization mass spectrometry (HPLC/UV-ESI-MS), allowing sensitive and precise identification of bioactive compounds in plant extracts. Chromatographic separation was carried out on an UltiMate 3000 HPLC system (Thermo Fisher Scientific) equipped with a reverse-phase C18 column (Merck, Darmstadt, Germany; 250 × 4 mm, 5 μm), maintained at 40 °C. Samples were kept at 5 °C prior to analysis. The mobile phase comprised solvent A (0.1% formic acid in water) and solvent B (0.1% formic acid in acetonitrile), applied under a gradient elution ranged from 2% to 95% B over 30 min. The flow rate was set at 1 mL/min, with an injection volume of 20 μL. Mass spectrometric detection was carried out in MS/MS (bbCID) mode using a Maxis Impact HD mass spectrometer (Bruker Daltonik) operating in negative ionization mode. The main operating parameters were as follows: a capillary voltage of 3000 V, a drying gas temperature of 200 °C, a flow rate of 8 L/min, a nebulizer pressure of 2 bar, and a plate offset of −500 V. Nitrogen served as both desolvation and nebulizing gas. UV detection was achieved using a diode array detector (Merck-Hitachi) over a wavelength range of 190–600 nm, with monitoring at 280, 320, and 360 nm, corresponding to different classes of phenolic compounds. Mass spectral data were acquired over an *m/z* range of 100–1,500 and processed using Chromeleon™ 7.2 software (Thermo Scientific) for compound identification (Drioiche et al., 2023).

#### 2.2.6 Evaluation of antioxidant activity

##### 2.2.6.1 Total antioxidant capacity

Total antioxidant capacity (TAC) of *C. ladanifer* aerial part extracts was evaluated using the phosphomolybdenum method (Hadi et al., 2024). In test tubes, 5 µL of each extract (25 mg/mL) was mixed with 1 mL of sulfuric acid (0.6 M), 1 mL of sodium phosphate (28 mM), and 1 mL of ammonium molybdate (4 mM). The mixture was incubated at 95 °C for 90 min and cooled at room temperature for 20–30 min. Absorbance was measured at 695 nm. TAC was expressed as milligrams of ascorbic acid equivalents per gram of dry extract (mg AAE/g DE), using ascorbic acid as the reference standard.

##### 2.2.6.2 DPPH assay

DPPH assay evaluates the ability of an extract to donate an electron to the synthetic radical DPPH* (2.2-diphenyl-1-picrylhydrazyl) and was performed according to Remok et al. (2023). In test tubes, increasing volumes of each extract, previously reconstituted in distilled water, were pipetted. Absolute ethanol was then added to obtain a volume of 200 μL, followed by the addition of 2.8 mL of ethanolic DPPH solution (24 μg/mL, w/v). After incubation in the dark for 30 min, absorbance was measured at 515 nm. The inhibition percentage was calculated using Equation 8:

$${\%}_{inhibition}=\left(\frac{A_{C}-A_{S}}{A_{C}}\right)\times 100$$ (8)

where A_C_: Absorbance of the negative control; A_S_: Absorbance of the sample.

##### 2.2.6.3 FRAP assay

Ferric reducing antioxidant power (FRAP) of the extracts was assessed following the method described by Saidi et al. (Moumen et al., 2025). In test tubes, increasing volumes of each extract were diluted to a final volume of 0.5 mL with distilled water and mixed with 2.5 mL of 0.2 M sodium phosphate buffer (pH 6.6) and 2.5 mL of 1% potassium ferricyanide. After incubation at 50 °C for 20 min, 2.5 mL of 10% trichloroacetic acid was introduced, followed by centrifugation for 10 min. Then, 2.5 mL of the supernatant was mixed with 2.5 mL of distilled water and 0.5 mL of a 0.1% ferric chloride solution. The absorbance was subsequently measured at 700 nm.

#### 2.2.7 Antimicrobial activity

Antimicrobial activity of the extract was evaluated by determining the minimum inhibitory concentration (MIC) using the broth microdilution method in 96-well microplates according to a previously reported protocol (Balouiri et al., 2016).

The extract stock solution was prepared in an ethanol/distilled water mixture (30:70, v/v). Serial dilutions were performed in wells 1–10 to obtain concentrations ranging from 75 to 0.1465 mg/mL in a final volume of 100 μL of Mueller–Hinton (MH) broth for bacterial strains and Sabouraud broth for fungal strains.

Subsequently, 100 μL of microbial inoculum was added to wells 1–11 to achieve final concentrations of approximately 10^6^ CFU/mL for bacterial strains and 10^4^ CFU/mL for fungal strains. Well 11 containing broth and inoculum served as the growth control, while well 12 containing only broth served as the sterility control. Gentamicin and terbinafine were used as positive controls for antibacterial and antifungal activities, respectively, while the solvent (ethanol/distilled water, 30:70, v/v) served as the negative control.

After incubation at 37 °C for 24 h, 10 μL of resazurin solution (6.75 mg/mL) was added to each well as an indicator of microbial growth, followed by an additional incubation for 2 h at 37 °C. A color change from blue-purple to pink indicated microbial growth. The MIC was defined as the lowest concentration of extract that prevented the color change.

To determine the minimum bactericidal (MBC) and fungicidal (MFC) concentrations, 10 μL aliquots from wells without visible growth was plated onto MH agar for bacterial strains or Sabouraud agar for fungal strains, then incubated for 24 h at 37 °C. The MBC or MFC was defined as the lowest concentration that completely inhibited colony formation on agar plates (Prasannabalaji et al., 2012).

The antimicrobial mode of action was assessed by determining the MBC/MIC or MFC/MIC ratio. Ratios ≤4 were considered indicative of bactericidal or fungicidal activity, whereas ratios >4 indicated bacteriostatic or fungistatic effects (National Committee for Clinical Laboratory Standards and Barry, 1999).

#### 2.2.8 Acute toxicity

Acute oral toxicity of the *C. ladanifer* aerial part aqueous extract was evaluated according to OECD guideline TG 423, with minor adaptations in the experimental design, including the use of mixed-sex groups (n = 6 per group) and intermediate dose levels for preliminary safety assessment (No, 2002). A total of 24 mice were used in the study. After a fasting period of 14 h, the animals were randomly divided into four groups (n = 6; ♂/♀ = 1:1).

The control group received distilled water (10 mL/kg), while the treated groups were orally administered single doses of the aqueous extract at doses of 0.5, 1, and 2 g/kg. Body weight was recorded at the beginning of the experiment.

Following administration, the animals were continuously observed for 10 h to detect any immediate signs of toxicity, and subsequently monitored daily for 14 days for behavioral changes, clinical symptoms, or mortality. This procedure provided preliminary information regarding the safety profile of the extract at the tested doses.

#### 2.2.9 Antihyperglycemic activity

Antihyperglycemic activity of the *C. ladanifer* aerial part aqueous extract was evaluated using the oral glucose tolerance test (OGTT) in rats, as previously described (Daoudi et al., 2020). Eighteen healthy rats were randomly divided into three groups (n = 6; ♂/♀ = 1:1): a control group receiving distilled water (10 mL/kg), a test group treated with the aqueous extract (400 mg/kg), and a reference group receiving glibenclamide (2 mg/kg). The dose of 400 mg/kg was selected as a preliminary screening dose based on previously reported pharmacological studies and supported by acute toxicity results indicating no toxicity up to 2000 mg/kg.

Baseline blood glucose levels were recorded before treatment (t_0_). After 30 min, an oral D-glucose load (2 g/kg) was administered to all animals, and blood glucose levels were subsequently measured at 30, 60, 90, and 150 min.

This experimental protocol allowed the evaluation of the effect of the extract on postprandial blood glucose regulation following glucose challenge.

#### 2.2.10 Statistical analysis

Results were statistically analyzed using analysis of variance (ANOVA), followed by Tukey’s *post hoc* test using GraphPad Prism software (version 9.5.1, GraphPad Prism software, San Diego, CA, United States of America) and they are shown as means and standard deviation. Statistics were considered to be significant at *p*-values of *p* < 0.05, *p* < 0.01, and *p* < 0.001.

#### 2.2.11 Molecular docking methodology

Molecular docking simulations were performed to investigate the interactions between the major phytochemical constituents identified in *Cistus ladanifer* aerial part extracts and selected protein targets associated with antioxidant, antimicrobial, and antihyperglycemic activities. Three crystallographic protein structures were retrieved from the Protein Data Bank (PDB): 3NVY, 1KZN, and 3TOP.

Protein 3NVY was selected as an antioxidant-related target because it contains a catalytic site involved in redox regulation and oxidative stress responses, making it suitable for evaluating the binding potential of phenolic compounds known for their radical-scavenging and electron-donating properties. Protein 1KZN corresponds to the ATP-binding domain of bacterial DNA gyrase B, an essential enzyme responsible for DNA supercoiling during replication and transcription. Inhibition of this enzyme disrupts bacterial DNA metabolism and has been widely exploited in the development of antibacterial agents. Protein 3TOP was selected as an antihyperglycemic target due to its involvement in glucose metabolism and glycemic regulation, providing a suitable model for investigating the molecular basis of the glucose-lowering effects observed experimentally.

The three-dimensional structures of the proteins were prepared using UCSF Chimera by removing crystallographic water molecules, co-crystallized ligands, and non-essential heteroatoms. Polar hydrogen atoms were added, and Gasteiger charges were assigned using AutoDock Tools (version 1.5.6). The prepared protein structures were subsequently saved in PDBQT format.

The molecular structures of the major compounds identified by HPLC/UV-ESI-MS analysis were obtained from the PubChem database and subjected to energy minimization using OpenBabel (version 3.0.1). The optimized ligands were converted into PDBQT format, and rotatable bonds were defined to allow conformational flexibility during docking simulations.

Docking calculations were performed using the AutoDock Vina molecular docking program (Trott and Olson, 2010). Grid boxes were centered on the active sites of the target proteins based on the coordinates of their co-crystallized ligands to ensure complete coverage of the binding pockets and key interacting residues. The exhaustiveness parameter was adjusted to provide adequate conformational sampling. For each ligand, multiple binding poses were generated, and the conformation exhibiting the lowest binding free energy (kcal/mol) was selected for further analysis.

The docking protocol was validated by re-docking the native co-crystallized ligand into the corresponding active site of each protein. The resulting root-mean-square deviation (RMSD) values confirmed the reliability of the docking procedure. Ligand–protein interactions, including hydrogen bonds, hydrophobic contacts, π–π stacking interactions, and electrostatic interactions, were analyzed and visualized using Discovery Studio Visualizer and PyMOL. Binding affinity values and interaction profiles were subsequently used to elucidate the potential molecular mechanisms underlying the antioxidant, antimicrobial, and antihyperglycemic activities of *C. ladanifer* extracts (Filali et al., 2024; Lahyaoui et al., 2024a; Lahyaoui et al., 2024b; Sghyar et al., 2024; Sghyar et al., 2025).

## 3 Results and discussion

### 3.1 Quality control of the plant material

Quality control parameters of *C. ladanifer* aerial parts are summarized in Table 3. The results showed a moisture content of 12.68% ± 0.42%, a pH of 4.16 ± 0.05, and an ash content of 5.50% ± 0.31%, indicating a well-dried and mineral-rich plant material of acceptable quality.

**TABLE 3:** Moisture content, pH, ash, and heavy metals present in *Cistus ladanifer* aerial part.

MC %	pH	Ash (%)	Heavy metal concentration (mg/g)						
			As	Zn	Co	Mn	Fe	Cu	Al
12.68 ± 0.42	4.16 ± 0.05	5.50 ± 0.31	0.066	1.18	ND	7.182	6.653	0.086	5.216

ND, not detected.

The elemental characterization results revealed the presence of arsenic (As), copper (Cu), zinc (Zn), aluminum (Al), iron (Fe), and manganese (Mn), while Cobalt (Co) was undetectable. Iron is crucial for chlorophyll synthesis, photosynthesis, and the prevention of chlorosis. Copper participates in enzymatic reactions and lignin formation, thereby contributing to structural support. Aluminum, in small amounts, aids in enzymatic processes and may regulate stomatal conductance (Abdelfattah et al., 2024). According to the literature, the elemental composition of *Cistus ladanifer* leaves is characterized by the presence of calcium, potassium, magnesium, iron, manganese, copper, and aluminum, whereas chromium, zinc, and nickel were not detected (Abdelfattah et al., 2024).

### 3.2 Phytochemical screening

Qualitative phytochemical analysis of the aerial parts of *C. ladanifer* revealed the presence of polysaccharides, proteins, reducing sugars, sterols, triterpenes, flavonoids, catechic tannins, leucoanthocyanins, saponosides, alkaloids, reducing compounds, and mucilages. In contrast, lipids, gallic tannins, anthocyanins, monosaccharides, and holosides were not detected. These findings indicate a chemically diverse phytochemical profile dominated by secondary metabolites commonly associated with biological activities. Similar results have been reported for aqueous extracts of *C. ladanifer* leaves, in which tannins, saponins, and flavonoids were also identified (Bousta et al., 2013). The occurrence of flavonoids, tannins, and saponosides may contribute to the antioxidant and antimicrobial activities investigated in the present study.

### 3.3 Extraction yields and phenolic compound contents

Extraction yields and phenolic compound contents of the aerial parts of *C. ladanifer* extracts are presented in Table 4. *CL1* exhibited the highest extraction yield (23.81% ± 0.63%), whereas *CL2* showed a lower yield (20.77% ± 0.84%).

**TABLE 4:** Extraction yields and total polyphenol, flavonoid, and condensed tannin contents of *Cistus ladanifer* aerial part extracts.

Extract	Extraction yield (%)	Total polyphenols (mg GAE/g DE)	Flavonoids (mg QE/g DE)	Condensed tannins (mg CE/g DE)
*CL1*	23.81 ± 0.63*	300.14 ± 1.26*	33.97 ± 1.32*	9.77 ± 0.25*
*CL2*	20.77 ± 0.84*	433.89 ± 1.82*	54.13 ± 0.52*	19.50 ± 0.87*

Values are expressed as mean ± standard deviation of triplicate determinations. Means were considered significantly different at *p < 0.05.

According to the literature, extraction yields of *C. ladanifer* aerial parts vary depending on the plant part and extraction technique employed. Aqueous Soxhlet extraction of the whole plant showed the highest yield (56.4%) compared with decocted and hydroethanolic Soxhlet extracts (El Karkouri et al., 2024). Maceration of the aerial parts with water and ethanol produced lower yields of 6.64% ± 0.06% and 5.67% ± 0.05%, respectively (Benali et al., 2020).

Regarding phenolic compounds, *CL2* showed the highest contents of total polyphenols, flavonoids, and condensed tannins, with values of 433.89 ± 1.82 mg GAE/g DE, 54.13 ± 0.52 mg QE/g DE, and 19.50 ± 0.87 mg CE/g DE, respectively. These differences may be related to solvent polarity and extraction conditions, which influence the solubility and recovery of phenolic constituents. Similar variability has been reported in previous studies, where aqueous maceration of aerial parts produced 76.98 ± 4.66 mg GAE/g extract (Benali et al., 2020), whereas hydroethanolic (70%) maceration of leaves yielded 520 ± 15 mg GAE/g extract (Köse et al., 2017). Ethanolic Soxhlet extraction of aerial leaves exhibited a total polyphenol content of 73.9 ± 8.7 µg GAE/mg DE (Bouothmany et al., 2022).

Overall, the quantitative differences observed between *CL1* and *CL2* indicate that extraction conditions substantially influenced both extraction efficiency and phenolic composition. The higher levels of polyphenols, flavonoids, and condensed tannins in *CL2* point to a distinct phenolic composition between the two extracts, which is further characterized in the following HPLC/UV-ESI-MS analysis and may contribute to the biological activities evaluated thereafter.

### 3.4 Chemical composition of selected extracts

HPLC/UV-ESI-MS profiling was subsequently performed to further characterize the chemical differences observed between the extracts. Chromatographic profiling enabled the identification of 40 compounds in *CL1* and 37 compounds in *CL2*. The corresponding HPLC chromatograms are presented in Figures 1, 2, respectively, while the chemical compositions of *CL1* and *CL2* are summarized in Tables 5 and 6.

**FIGURE 1:**
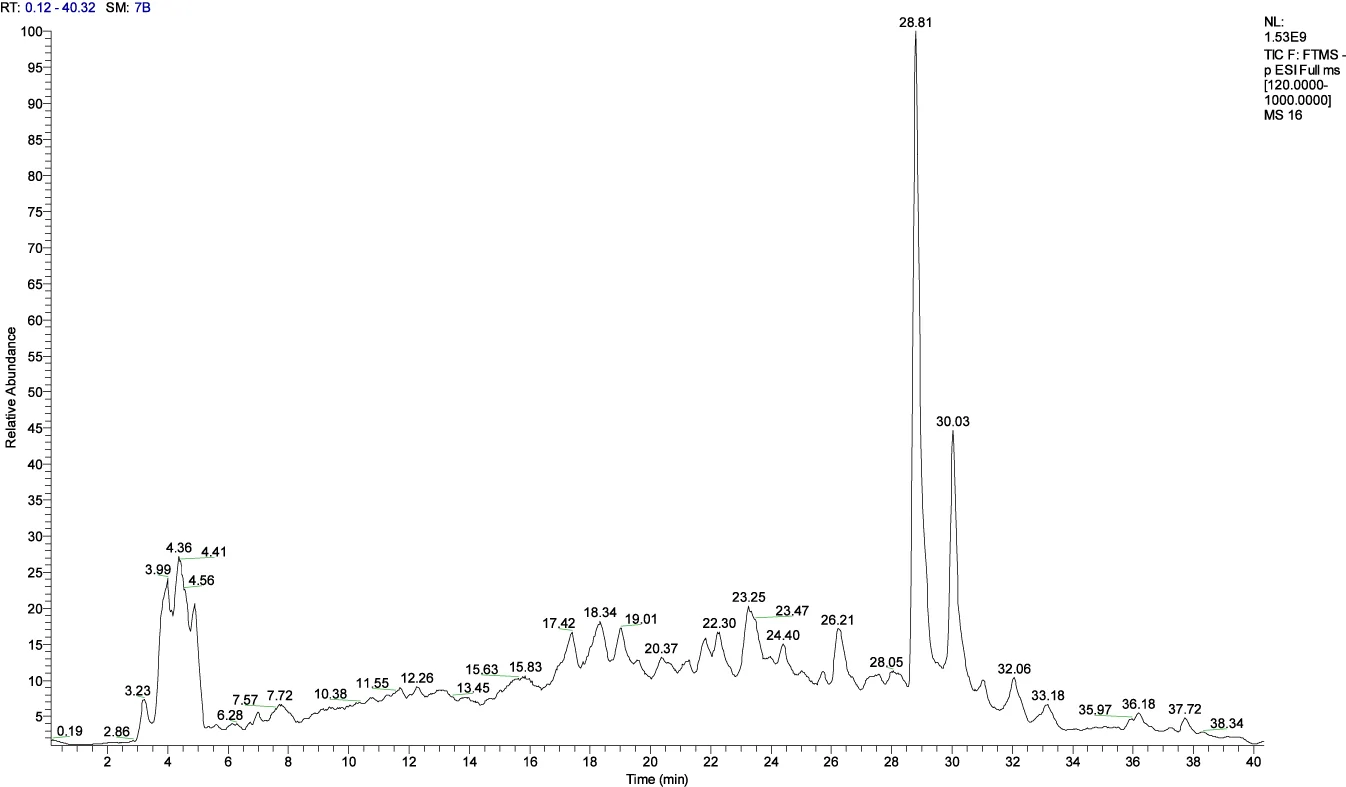
HPLC chromatogram of *CL1* of *Cistus ladanifer* aerial parts.

**FIGURE 2:**
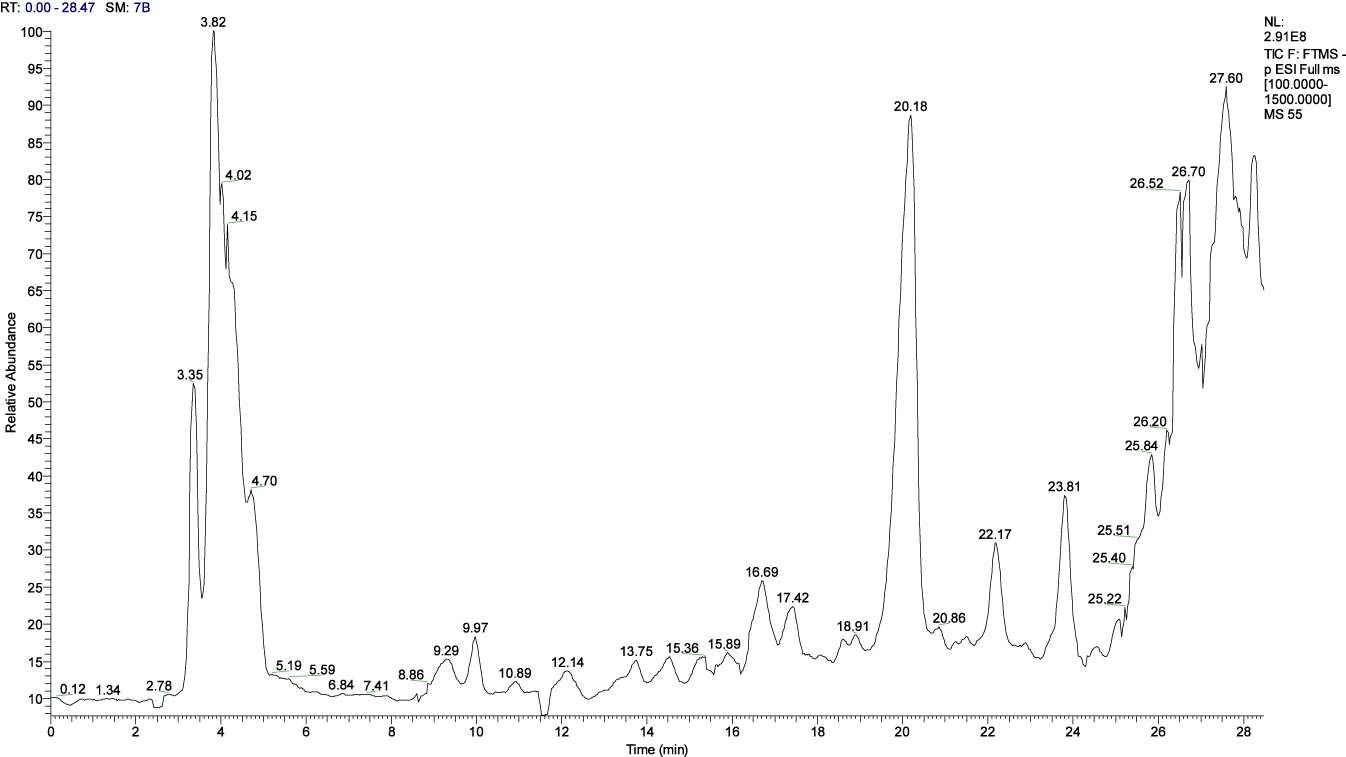
HPLC chromatogram of *CL2* of *Cistus ladanifer* aerial parts.

**TABLE 5:** Chemical composition of *CL1* of *Cistus ladanifer* aerial parts.

N°	RT (min)	Area (%)	(m/z)	MW	Identified compound	Molecular formula	Class
1	4.33	1.41	333.1/667	334	Amaronol B	C_16_H_14_O_8_	Other phenolic compounds
2	4.55	1.65	481.1/963	482	Silibinin	C_25_H_22_O_10_	Flavonoid
3	4.65	0.91	245.0/489.1	246	Hispidin	C_13_H_10_O_5_	Polyphenols
4	4.86	3.97	293.1	294	Embelin	C_17_H_26_O_4_	Others (quinone)
5	5.97	1.26	191.1	192	Quinic acid	C_7_H_12_O_6_	Others (organic acid)
6	6.16	0.29	387.1	388	Ethyl rosmarinate	C_20_H_20_O_8_	Other phenolic compounds
7	6.85	1.81	331.1	332	Galloyl glucose	C_13_H_16_O_10_	Polyphenols
8	7.50	2.85	169.0	170	Gallic acid	C_7_H_6_O_5_	Phenolic acid
9	9.18	6.09	783.1/469	784	Leonoside B	C_36_H_48_O_19_	Other phenolic compounds
10	10.61	9.49	541.0	542	Resveratrol 4'-(6-galloylglucoside)	C_27_H_26_O_12_	Polyphenols
11	12.80	18.44	285	286	Kaempferol	C_15_H_10_O_6_	Flavonoid
12	15.02	7.21	625.0	626	Quercetin 34′-diglucoside	C_27_H_30_O_17_	Flavonoid
13	15.70	1.4	489.2	490	Kaempferol 3-(6″-acétylglucoside)	C_23_H_22_O_12_	Flavonoid
14	15.99	1.72	483.1	484	Digalloyl glucose	C_20_H_20_O_14_	Tannin
15	16.50	0.79	515	516	Isochlorogenic acid A	C_25_H_24_O_12_	Phenolic acid
16	16.98	1.28	369.1	370	Ferulic acid -4′-O-glucuronide	C_16_H_18_O_10_	Phenolic acid
17	17.28	1.1	421.2	422	Catechin 7-O-apiofuranoside	C_20_H_22_O_10_	Flavonoid
18	18.08	2.44	197.0	198	Syringic acid	C_9_H_10_O_5_	Phenolic acid
19	18.97	4.57	469.0	470	Valoneic acid dilactone	C_21_H_10_O_13_	Tannin
20	19.95	1.64	377.2	378	Oleuropein aglycon	C_19_H_22_O_8_	Others (iridoid)
21	20.67	4.57	465.1	466	Taxifolin 7-glucoside	C_21_H_22_O_12_	Flavonoid
22	22.04	2.24	609.1	610	Rutin	C_27_H_30_O_16_	Flavonoid
23	22.47	1.06	477.1/955.1	478	Quercetin 7-glucuronide	C_21_H_18_O_13_	Flavonoid
24	22.91	1.58	273	274	3,4,4′,7-tetrahydroxyflavan	C_15_H_14_O_5_	Flavonoid
25	23.45	3.24	301	302	Quercetin	C_15_H_10_O_7_	Flavonoid
26	23.83	1.16	417.2	418	Kaempferol 3-*O*-arabinoside	C_20_H_18_O_10_	Flavonoid
27	24.14	0.54	447.1/895.1	448	Kaempferol-7-O-glucoside	C_21_H_20_O_11_	Flavonoid
28	24.37	1.77	461.1	462	Isorhamnetin 7-rhamnoside	C_22_H_22_O_11_	Flavonoid
29	24.86	1.41	429.2/859.4	430	α-tocopherol	C_29_H_50_O_2_	Others (terpenoid)
30	25.33	0.82	491.2	492	Isosalvianolic acid C	C_26_H_20_O_10_	Phenolic acid
31	25.64	1.1	527.2	528	Tremulacin	C_27_H_28_O_11_	Other phenolic compounds
32	26.15	4.67	593.1	594	Kaempferol-3-O-rutinoside	C_27_H_30_O_15_	Flavonoid
33	26.95	0.9	265.1	266	7-Methoxy-2-methylisoflavone	C_17_H_14_O_3_	Flavonoid
34	27.23	0.67	269.0	270	Apigenin	C_15_H_10_O_5_	Flavonoid
35	28.31	0.85	299.1	300	Kaempferide	C_16_H_12_O_6_	Flavonoid
36	29.01	0.7	739.2	740	Kaempferol 3-O-(4″,6″-O-di-p-coumaroyl)-glucoside	C_39_H_32_O_15_	Flavonoid
37	29.81	0.6	283.1	284	Glycitein	C_16_H_12_O_5_	Flavonoid
38	31.8	0.55	319.2	320	Dihydromyricetin	C_15_H_12_O_8_	Flavonoid
39	35.2	0.67	345	346	Rosmanol	C_20_H_26_O_5_	Other phenolic compounds
40	36.16	0.54	327.3	328	Salvigenin	C_18_H_16_O_6_	Flavonoid

**TABLE 6:** Chemical composition of *CL2* of *Cistus ladanifer* aerial parts.

N°	RT (min)	Area (%)	(m/z)	MW	Identified compound	Molecular formula	Class
1	4.03	1.18	317	318	Myricetin	C_15_H_10_O_8_	Flavonoid
2	4.20	1.51	327	328	Salvigenin	C_18_H_16_O_6_	Flavonoid
3	4.40	7.17	179	180	Caffeic acid	C_9_H_8_O_4_	Phenolic acid
4	4.57	3.87	481	482	Silibinin	C_25_H_22_O_10_	Flavonoid
5	4.73	5.13	401	402	Flavanone 7-O-glucoside	C_21_H_22_O_8_	Flavonoid
6	5.79	0.1	293	294	Embelin	C_17_H_26_O_4_	Others (quinone)
7	6.30	0.13	225	226	3-Flavanol	C_15_H_14_O_2_	Flavonoid
8	8.84	0.78	295	296	p-Coumaroyltartaric acid	C_13_H_12_O_8_	Phenolic acid
9	9.28	3.86	169	170	Gallic acid	C_7_H_6_O_5_	Phenolic acid
10	9.93	2.23	331	332	Galloyl glucose	C_13_H_16_O_10_	Polyphenols
11	10.47	1.86	499	500	Taxifolin 7-O-rhamnoside	C_21_H_22_O_11_	Flavonoid
12	10.82	5.33	781	782	Punicalin (isomer 1)	C_34_H_22_O_22_	Tannin
13	11.38	2.48	283	284	Glycitein	C_16_H_12_O_5_	Flavonoid
14	11.77	2.91	191	192	Quinic acid	C_7_H_12_O_6_	Others (organic acid)
15	12.15	4.52	183	184	Methyl gallate	C_8_H_8_O_5_	Phenolic acid
16	13.40	7.2	469	470	Valoneic acid dilactone	C_21_H_10_O_13_	Tannin
17	13.66	8.5	285	286	Kaempferol	C_15_H_10_O_6_	Flavonoid
18	14.17	3.36	483.1	484	Gallic acid 4-O-(6-galloylglucoside)	C_20_H_20_O_14_	Phenolic acid
19	14.50	7.8	625/1251	626	Quercetin 3,4′-diglucoside	C_27_H_30_O_17_	Flavonoid
20	15.13	10.68	297	298	Apigenin 7,4′-dimethyl ether	C_17_H_14_O_5_	Flavonoid
21	15.84	6.64	541/1083.1	542	Resveratrol 4'-(6-galloylglucoside)	C_27_H_26_O_12_	Polyphenols
22	17.10	1.56	489.2	490	Kaempferol 3-(6″-acetylglucoside)	C_23_H_22_O_12_	Flavonoid
23	17.47	0.4	431	432	Kaempferol 7-rhamnoside	C_21_H_20_O_10_	Flavonoid
24	18.63	0.13	361.1/723.3	362	Ligstroside aglycone	C_19_H_22_O_7_	Other phenolic compounds
25	19.21	0.07	373	374	Methyl rosmarinate	C_19_H_18_O_8_	Phenolic acid
26	20.16	1.84	197	198	Syringic acid	C_9_H_10_O_5_	Phenolic acid
27	21.44	1.2	301/603	302	Quercetin	C_15_H_10_O_7_	Flavonoid
28	22.29	0.13	533.2	534	Amurensin	C_26_H_30_O_12_	Flavonoid
29	23.38	0.29	355	356	Ferulic acid 4-O-glucoside	C_16_H_20_O_9_	Phenolic acid
30	23.73	0.19	447/895	448	Kaempferol-O-hexoside	C_21_H_20_O_11_	Flavonoid
31	24.09	0.35	417	418	Kaempferol 3-*O*-arabinoside	C_20_H_18_O_10_	Flavonoid
32	24.59	0.1	359	360	Rosmarinic acid	C_18_H_16_O_8_	Phenolic acid
33	25.25	1.34	367	368	3-Feruloylquinic acid	C_17_H_20_O_9_	Phenolic acid
34	25.41	1.01	339	368	Salvianolic acid G	C_18_H_12_O_7_	Phenolic acid
35	25.75	2.83	593/1,187	340	Kaempferol 3-rutinoside	C_27_H_30_O_15_	Flavonoid
36	26.43	0.48	265	266	7-Methoxy-2-methylisoflavone	C_17_H_14_O_3_	Flavonoid
37	26.67	0.4	311.1/623.3	312	Caffeoyl tartaric acid	C_13_H_12_O_9_	Phenolic acid

Compound characterization was achieved through the combined evaluation of retention time and mass spectrometric data, supported by comparison with previously reported literature. The detected constituents consisted mainly of phenolic acids, flavonoids, and related bioactive compounds, representing more than 99% of the extracts’ total composition.

The distribution of chemical classes in *CL1* and *CL2*, shown in Figure 3, revealed that flavonoids were the predominant class in both extracts, with a slightly higher proportion in *CL1*, whereas *CL2* was markedly enriched in phenolic acids and tannins compared with *CL1*. Conversely, *CL1* showed a higher contribution of polyphenols and other phenolic compounds, the latter being nearly absent in *CL2*. These class-level differences may partly explain the distinct biological activities observed for *CL1* and *CL2* in the subsequent assays.

**FIGURE 3:**
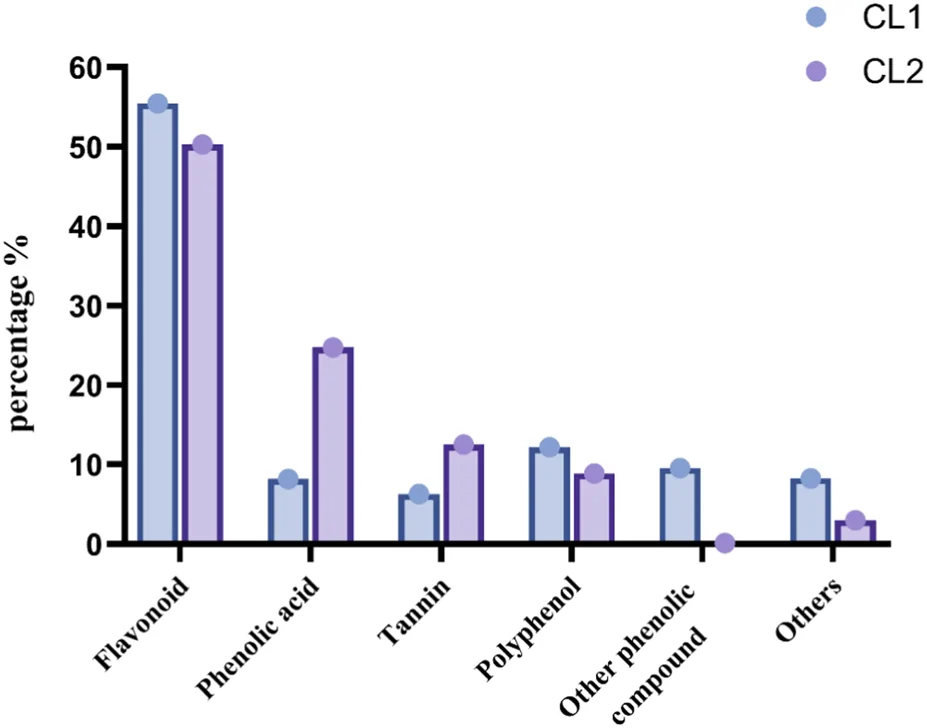
Percentage distribution of identified compound classes in *Cistus ladanifer* aerial part extracts.

For a relative abundance equal to or greater than 5%, *CL1* revealed four compounds, namely, leonoside B, resveratrol 4'-(6-galloylglucoside), kaempferol, and quercetin 3,4′-diglucoside, whereas *CL2* revealed eight compounds, namely, caffeic acid, flavanone 7-O-glucoside, punicalin, valoneic acid dilactone, kaempferol, quercetin 3,4′-diglucoside, apigenin 7,4′-dimethyl ether, and resveratrol 4'-(6-galloylglucoside). Figure 4 illustrates the chemical structures of these major compounds.

**FIGURE 4:**
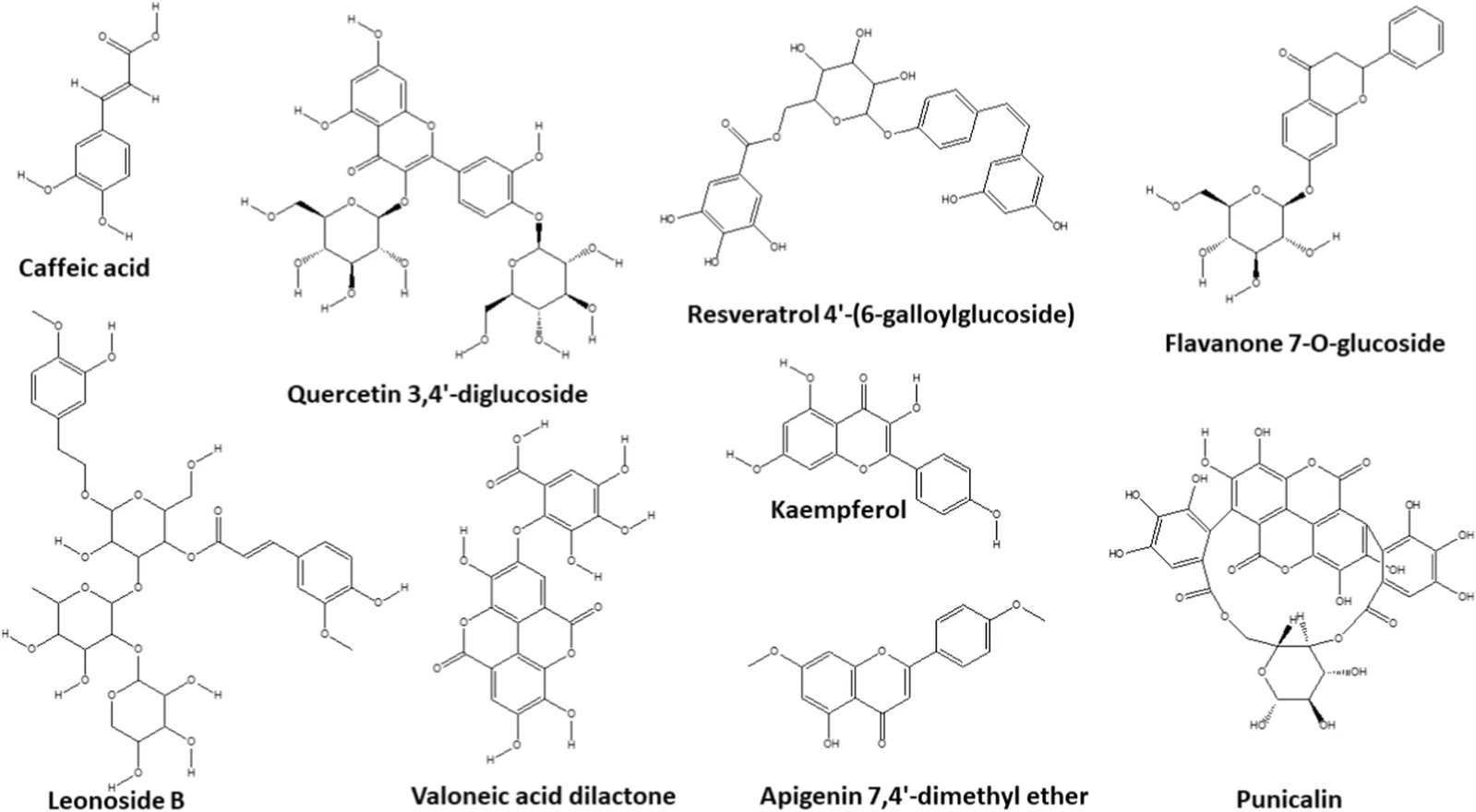
Structures of the major compounds identified in *Cistus ladanifer* aerial part extracts.

Phenolic acids and flavonoids are widely recognized for their antioxidant, anti-inflammatory, cardioprotective, antimicrobial, antiviral, antidiabetic, and anticancer properties (Al-Khayri et al., 2022; Saleem et al., 2022). Caffeic acid exhibits antibacterial (against *S. aureus*, *S. pneumoniae*, *E. coli*, *K. pneumoniae*, and *P. aeruginosa*), antioxidant, anti-inflammatory, antidiabetic, hepatoprotective, and antiviral activities (Bhattacharya and Mandal, 2025). Kaempferol exhibits cardioprotective, anti-inflammatory, antidiabetic, antioxidant, antimicrobial, and anticancer effects (Dong et al., 2025), whereas punicalin shows antioxidant and antihepatotoxic activities (Lin et al., 2001). Silibinin has potential clinical value in diabetes mellitus, neurodegenerative disorders, and cancers (Wing Ying Cheung et al., 2010). Gallic acid and methyl gallate display strong antioxidant, anti-inflammatory, antimicrobial, and antitumor activities (Liang et al., 2023; Verma et al., 2013). Embelin possesses analgesic, anticancer, and anti-inflammatory activities (Basha et al., 2022), while quercetin contributes to anti-carcinogenic, anti-inflammatory, antiviral, and antioxidant effects (Li et al., 2016). Compounds such as catechin, quercetin, kaempferol, gallic acid, and resveratrol exhibit anti-inflammatory, and antioxidant activities (da Silva, 2021). Valoneic acid dilactone strongly inhibits xanthine oxidase, surpassing allopurinol (Unno et al., 2004). In the present study, these compounds were detected at different relative abundances in *CL1* and *CL2*, suggesting that their distribution pattern, rather than their mere presence, may contribute to the biological responses observed.

Previous phytochemical investigations of *C. ladanifer* aerial parts have also reported the presence of numerous phenolic acids, ellagitannins, flavonoids, and phenolic glycosides, including gallic acid, punicalin, quercetin derivatives, kaempferol derivatives, ellagic acid derivatives, and apigenin derivatives (Fernández-Arroyo et al., 2010). The occurrence of several of these compounds in the present study further supports the richness of *C. ladanifer* aerial parts in phenolic constituents and confirms previous reports on the phytochemical composition of this species.

Given the recognized antioxidant, antimicrobial, and antidiabetic properties of several compounds identified in *CL1* and *CL2*, particularly flavonoids and phenolic acids, the chemical profiles obtained provide a plausible basis for interpreting the biological activities evaluated in the following sections.

### 3.5 Antioxidant activities

Based on the differences observed in phenolic contents and chemical profiles, the antioxidant potential of the two extracts was evaluated using TAC, DPPH, and FRAP assays (Table 7).

**TABLE 7:** Antioxidant activities of *Cistus ladanifer* aerial part extracts by TAC, DPPH, FRAP assays.

Extract	TAC (mg EAA/g DE)	DPPH* IC_50_ (µg/mL)	FRAP EC_50_ (µg/mL)
*CL1*	364.18 ± 0.83*	7.45 ± 0.00*	10.43 ± 0.59*
*CL2*	462.04 ± 0.41*	7.06 ± 0.31*	10.42 ± 0.83*
Ascorbic acid	-	6.39 ± 0.61*	6.27 ± 0.72*

Values are expressed as mean ± standard deviation of triplicate determinations. Means were considered significantly different at *p < 0.05.

*CL2* exhibited the highest antioxidant activity, with a TAC of 462.04 ± 0.41 mg EAA/g DE and a DPPH IC_50_ of 7.06 ± 0.31 μg/mL. In contrast, no significant difference was observed between *CL1* and *CL2* in the FRAP assay, with EC_50_ values of 10.43 ± 0.59 and 10.42 ± 0.83 μg/mL, respectively. Ascorbic acid, used as the reference standard, showed stronger activity in both DPPH and FRAP assays (IC_50_ = 6.39 ± 0.61 μg/mL; EC_50_ = 6.27 ± 0.72 μg/mL).

The higher TAC and DPPH activity of *CL2* are consistent with its higher total polyphenol, flavonoid, and condensed tannin contents (Table 4) and its distinct phenolic profile revealed by HPLC/UV-ESI-MS. Phenolic acids and flavonoids may contribute to antioxidant activity through hydrogen donation and electron-transfer mechanisms. However, the present results do not allow the contribution of individual compounds to be established, and the observed activity is likely related to the combined effects of the phenolic constituents.

Previous studies have reported a DPPH IC_50_ of 54 μg/mL for a hydroethanolic Soxhlet extract of the whole plant, whereas a decocted extract showed FRAP activity with an EC_50_ of 0.48 mg/mL (El Karkouri et al., 2024). An ethanolic Soxhlet extract of aerial leaves was also found to exhibit a DPPH IC_50_ of 266.6 ± 0.83 μg/mL (Bouothmany et al., 2022). The stronger antioxidant activity observed in the present study may therefore reflect differences in plant part, extraction procedure, solvent system, and the resulting phenolic composition.

### 3.6 Antimicrobial activity

The antimicrobial activity of the extracts was subsequently investigated to determine whether the differences in chemical composition observed between *CL1* and *CL2* were also reflected in their activity against bacterial and fungal strains (Table 8).

**TABLE 8:** MIC, MBC, and MFC (mg/mL) values of *Cistus ladanifer* aerial part extracts, together with the MIC values of the reference antibiotic and antifungal agents.

Strain	*CL1*			*CL2*			Gentamicin	Terbinafine
	MIC (mg/mL)	MBC or MFC (mg/mL)	Ratio	MIC (mg/mL)	MBC or MFC (mg/mL)	Ratio	MIC (mg/mL)	MIC (mg/mL)
*S. epidermidis*	18.75	>75	-	9.38	75	8	2	​
*E. coli* ESBL	18.75	>75	-	9.38	>75	-	2	​
*K. pneumoniae*	18.75	75	4	18.75	75	4	≤1	​
*E. coli*	18.75	75	4	9.38	>75	-	2	​
*P. mirabilis*	18.75	75	4	18.75	75	4	2	​
*P. aeruginosa*	18.75	>75	-	18.75	>75	-	2	​
*E. cloacae*	18.75	>75	-	9.38	>75	-	>4	​
*S. aureus* BLACT	18.75	9.38	0.5	18.75	9.38	2	<0.5	​
*S. agalactiae* (B)	37.5	75	2	37.5	75	2	≤250	​
*C. kefyr*	4.69	>75	-	4.69	>75	-	​	25
*S. cerevisiae*	18.75	>75	-	4.69	>75	-	​	3.13
*A. niger*	37.5	75	2	37.5	9.38	0.25	​	3.13
*C. tropicalis*	18.75	>75	-	37.5	>75	-	​	12.5
*C. dubliniensis*	9.38	>75	-	9.38	>75	-	​	3.13
*C. albicans*	18.75	>75	-	18.75	>75	-	​	12.5
*C. krusei*	>75	>75	-	18.75	>75	-	​	50
*C. parapsilosis*	18.75	>75	-	18.75	>75	-	​	6.25

The lowest MIC value (4.69 mg/mL) was recorded for *CL1* against *C. kefyr*, and for *CL2* against *C. kefyr* and *S. cerevisiae*. At an MIC of 9.38 mg/mL, *CL2* inhibited *S. epidermidis, E. coli* ESBL, *E. coli*, *Enterobacter cloacae,* and *C. dubliniensis*, whereas *CL1* inhibited *C. dubliniensis*. Overall, *CL2* showed greater activity against several tested microorganisms, whereas identical MIC values were observed for both extracts against several strains including *K. pneumoniae*, *Proteus mirabilis*, *P. aeruginosa*, *S. aureus* BLACT, *Streptococcus agalactiae* (B), *C. kefyr*, *A. niger*, *C. dubliniensis*, *C. albicans*, and *C. parapsilosis*, while MIC values differed between the two extracts for the remaining strains.

Based on the MBC/MIC and MFC/MIC ratios, *CL1* showed bactericidal activity against *K. pneumoniae, E. coli, P. mirabilis, S. aureus* BLACT, and *S. agalactiae* (B), and fungicidal activity against *A. niger*. *CL2* exerted bactericidal activity against *K. pneumoniae, P. mirabilis, S. aureus* BLACT, and *S. agalactiae* (B), and fungicidal activity against *A. niger*.

The antimicrobial findings can be considered in relation to the chemical profiles presented above. Both extracts contained numerous flavonoids and phenolic derivatives, although their relative distribution differed: *CL1* was characterized by a higher abundance of kaempferol and quercetin derivatives, whereas *CL2* showed higher levels of valoneic acid dilactone and apigenin derivatives, as well as the presence of compounds not detected in *CL1*, such as caffeic acid and punicalin. These compounds and related phenolic classes have been associated with antimicrobial effects through several mechanisms, including disruption of microbial membranes, alteration of membrane permeability, inhibition of essential enzymes, metal chelation, and interference with nucleic acid synthesis (Daglia, 2012; Farhadi et al., 2019). The higher relative abundance of several of these constituents in *CL2* may therefore contribute to its stronger activity against some of the tested microorganisms.

Differences in susceptibility among the tested strains may also be related to differences in their cellular structures and the accessibility of cellular targets to bioactive compounds. In bacteria, structural differences in the cell envelope, particularly the outer membrane of Gram-negative species, may influence the penetration and activity of phenolic constituents. Similarly, differences in the susceptibility of the tested fungal strains may reflect variations in their cellular composition and sensitivity to phenolic compounds.

Previous studies reported MIC values of ethanolic macerates of the aerial parts, determined by the agar disc diffusion method, of 3.12 mg/mL for *Clavibacter michiganensis* subsp. *Michiganensis* and 6.25 mg/mL for *Pseudomonas savastanoi* pv. *savastanoi* (Benali et al., 2020). Using the microdilution assay, ethanolic macerates of the aerial parts showed MIC values of 25, 1.56, and 0.78 mg/mL for *E. coli*, *S. aureus*, and *S. epidermidis*, respectively (Köse et al., 2017). In another study, agar dilution showed that acetone/water, methanol, and ethanol crude extracts of the whole plant exhibited the highest activity against *K. pneumoniae* and *B. cereus*, with MIC values ranging from 0.625 to 1.25 mg/mL (Fe et al., 2012).

Because the extracts contain multiple constituents, synergistic or additive interactions among compounds and other constituents cannot be excluded.

### 3.7 Acute toxicity

Following the chemical characterization and assessment of biological activities, the acute oral toxicity of *CL2*, which exhibited the highest phenolic content and showed stronger responses in several of the biological assays, was evaluated *in vivo*. This assessment was conducted to determine whether the extract could produce observable acute toxic effects following oral administration and to provide an initial safety profile under the experimental conditions used.

Oral administration of *CL2* at 2 g/kg did not result in mortality or observable clinical signs of toxicity, including diarrhea, vomiting, or abnormal mobility, throughout the monitoring period. These findings indicate that *CL2* showed no evidence of acute oral toxicity at the tested dose under the experimental conditions. Previous studies have similarly reported a lack of acute toxicity for *C. ladanifer* preparations at lower doses. An acute toxicity study of a leaf decoction reported no behavioral abnormalities or morphological changes at doses of 150, 175, and 200 mg/kg (Bousta et al., 2013). Likewise, a study of *C. ladanifer* leaf infusion reported no mortality or apparent toxic effects at doses below 2000 mg/kg in mice, whereas higher doses of 3000–5000 mg/kg induced signs of toxicity and mortality rates of up to 30% (El Kabbaoui et al., 2017). These findings suggest that the acute toxicity of *C. ladanifer* preparations may be dose-dependent and may also vary according to the plant material and extraction procedure.

Taken together, the absence of observable acute toxicity at 2 g/kg, combined with the antioxidant and antimicrobial activities observed for *CL2*, provides an initial basis for considering this extract for further biological investigation. However, additional studies, including repeated-dose toxicity and more detailed safety assessments, are required before definitive conclusions regarding its overall safety can be drawn.

### 3.8 Antihyperglycemic effect

Given the favorable biological profile and the absence of observable acute toxicity for *CL2*, its potential antihyperglycemic effect was subsequently investigated using an oral glucose tolerance test (OGTT).

Changes in blood glucose levels in control rats and in rats treated with glibenclamide or *CL2* of *C. ladanifer* aerial parts during the oral glucose tolerance test (OGTT) are shown in Figure 5A. Following glucose administration, the control group showed a marked increase in glycemia, reaching a maximum at 60 min, reflecting postprandial hyperglycemia. In contrast, rats treated with glibenclamide and *CL2* exhibited a significantly lower rise in blood glucose levels at 60 min. At 90 min, a slight increase in glycemia was observed in glibenclamide-treated rats, whereas a marked decrease was noted in rats treated with *CL2*, indicating that *CL2* attenuated the glycemic peak. At 150 min, blood glucose levels tended to return toward near-basal values in all groups. The area under the curve (AUC) of the OGTT (Figure 5B) was significantly reduced in both the glibenclamide- and *CL2*-treated groups compared with the control group, indicating improved glucose tolerance.

**FIGURE 5:**
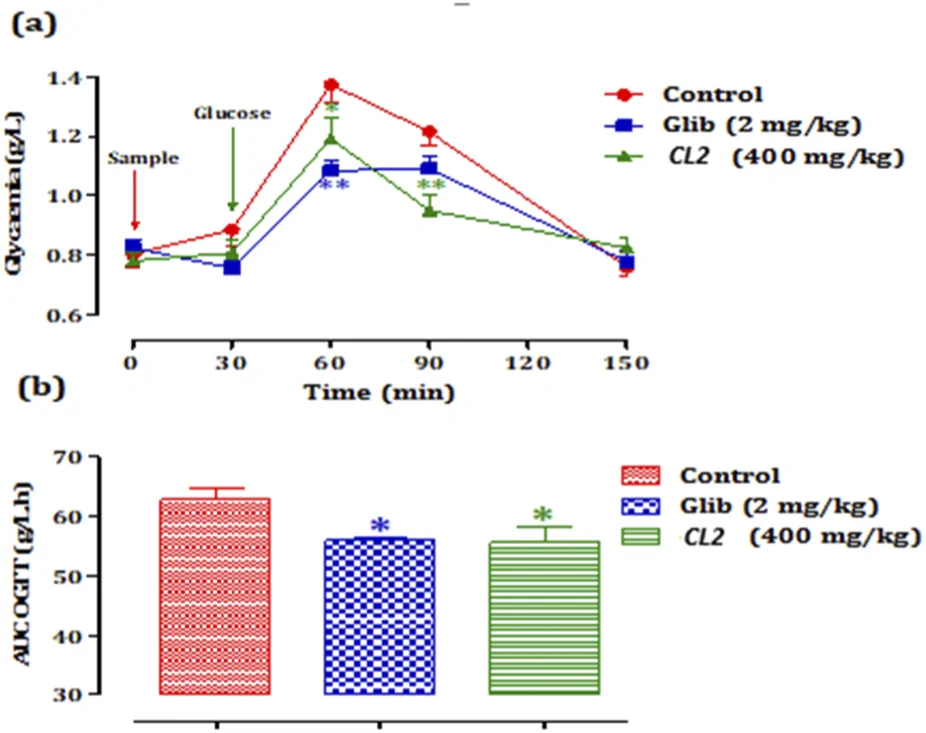
Postprandial blood glucose **(a)** and area under the postprandial curve **(b)** in normal rats after administration of *Cistus ladanifer* aerial part extract (*CL2*) and glibenclamide. Data are expressed as mean ± SEM (n = 6). Statistical significance: ***p < 0.001; **p < 0.01; *p < 0.05: compared with the control group.

The antihyperglycemic response observed for *CL2* may be related, at least in part, to its phenolic and flavonoid composition identified by HPLC/UV-ESI-MS. The higher phenolic and flavonoid contents observed for *CL2* (Table 4), together with the presence of several potentially bioactive constituents (Table 6), provide a plausible chemical basis for the observed effect. Nevertheless, the present data do not allow the contribution of individual compounds or possible synergistic interactions to be established, and further mechanistic investigations are required.

### 3.9 Molecular docking study

To further explore the possible molecular mechanisms underlying the biological activities observed experimentally, a molecular docking study was carried out between the main compounds identified in *Cistus ladanifer* aerial part extracts and the selected protein targets involved in antioxidant, antimicrobial, and antihyperglycemic processes. Docking simulations were performed to evaluate the binding affinity and interaction patterns between the phytochemicals and the active sites of the proteins.

#### 3.9.1 Antioxydant activity

Molecular docking analysis revealed significant interactions between the major phenolic compounds identified in *CL1* and *CL2* of *C. ladanifer* aerial parts and the antioxidant-related target protein (3NVY). The binding affinities ranged from −3.88 to −5.89 kcal/mol, indicating a moderate to strong interaction between the ligands and the receptor active site (Table 9).

**TABLE 9:** Docking score and interaction between the compounds and 3NVY protein.

Name	Binding energy (kcal/mol)	Receptor	Interaction	Distance
Caffeic acid	−3.88	CYS 113	H-donor	4.18
Flavanone 7-O-glucoside	−4.82	GLU 45	H-donor	2.78
		ARG 149	pi-H	4.69
Punicalin isomer 1	−5.4	CYS 148	H-acceptor	3.68
		ARG 149	pi-H	3.82
Valoneic acid dilactone	−5.12	CYS 48	H-donor	3.62
		CYS 113	H-donor	3.78
		CYS 148	H-acceptor	3.39
		ARG 149	pi-H	3.96
Kaempferol	−4.65	GLU 45	H-donor	3.12
Quercetin 3,4′-diglucoside	−5.43	GLU 45	H-donor	2.91
		GLY 47	H-acceptor	3.68
Apigenin 7,4′-dimethyl ether	−4.55	CYS 148	H-acceptor	3.42
Resveratrol 4'-(6-galloylglucoside)	−5.79	CYS 150	H-donor	3.42
Leonoside B	−5.89	CYS 113	H-donor	4.24
		GLU 45	H-donor	2.92
		GLY 47	H-acceptor	3.61
		GLY 47	H-acceptor	3.45

Among the tested compounds, leonoside B (−5.89 kcal/mol) and resveratrol 4'-(6-galloylglucoside) (−5.79 kcal/mol) exhibited the most favorable binding energies, suggesting a higher stability of the ligand–protein complexes. Leonoside B established multiple hydrogen-bond interactions with key amino acid residues, including CYS113, GLU45, and GLY47, which may enhance the stabilization of the ligand within the catalytic pocket (Figure 6). Similarly, resveratrol 4'-(6-galloylglucoside) interacted with residues such as CYS150 through hydrogen bonding, contributing to its strong binding affinity.

**FIGURE 6:**
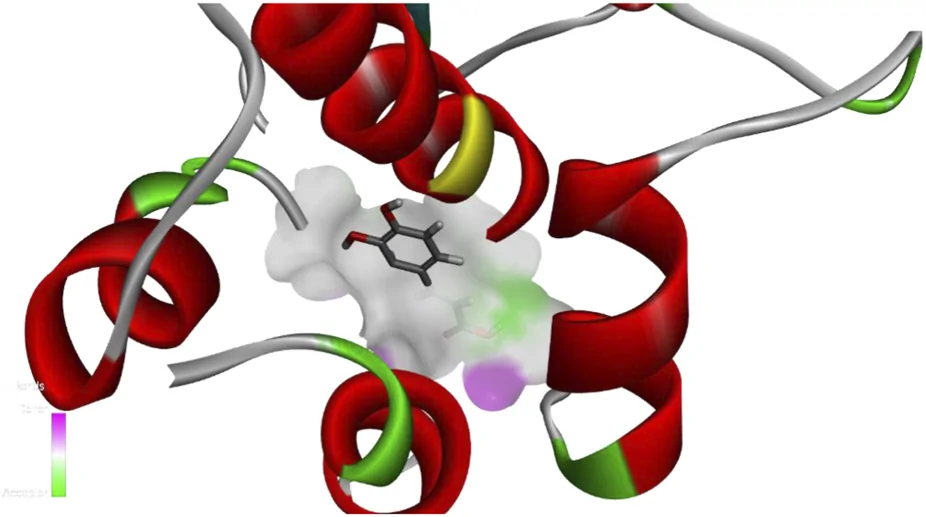
Docking of leonoside B and 3NVY protein.

Other phenolic compounds, including punicalin isomer 1 (−5.4 kcal/mol) and valoneic acid dilactone (−5.12 kcal/mol), also demonstrated significant interactions involving hydrogen bonds and π–H interactions with residues such as ARG149 and CYS148. These interactions are known to contribute to ligand stabilization and may facilitate electron transfer mechanisms involved in antioxidant activity. These findings are consistent with the high antioxidant activity observed experimentally for the phenolic-rich extracts of *C. ladanifer*. Phenolic compounds are widely recognized for their ability to neutralize reactive oxygen species through hydrogen donation and resonance stabilization mechanisms. The strong docking scores obtained for leonoside B and resveratrol derivatives support their potential role as key contributors to the antioxidant capacity of the plant extracts *CL1* and *CL2*.

When compared with well-known antioxidant molecules such as ascorbic acid, several compounds identified in *C. ladanifer* exhibited comparable binding patterns with the target protein. The presence of multiple hydroxyl groups in these phenolic structures enhances their ability to establish hydrogen-bond interactions with active site residues, thereby promoting radical scavenging activity.

#### 3.9.2 Antimicrobial activity

Docking simulations performed against the microbial target protein (1KZN) showed binding energies ranging from approximately −4.0 to −6.54 kcal/mol (Table 10).

**TABLE 10:** Docking score and interaction between the compounds and 1KZN protein.

Compound	Binding energy (kcal/mol)	Receptor	Interaction	Distance
Caffeic acid	3.89	CYS 113	H-donor	4.18
Flavanone 7-O-glucoside	−5.09	GLU 45	H-donor	3.34
Punicalin isomer 1	−5.4	CYS 148	H-acceptor	3.68
		ARG 149	pi-H	3.82
Valoneic acid dilactone	−4.89	GLY 42	H-donor	2.81
Kaempferol	−4.65	GLU 45	H-donor	3.13
Quercetin 3,4′-diglucoside	−5.86	CYS 148	H-donor	2.98
		ARG 149	H-acceptor	2.97
		ARG 149	H-acceptor	3.19
		ARG 149	H-acceptor	3.01
Apigenin 7,4′-dimethyl ether	−4.55	CYS 148	H-acceptor	3.42
Resveratrol 4'-(6-galloylglucoside)	−5.82	CYS 148	H-donor	2.96
Leonoside B	−6.54	ARG 149	H-acceptor	3.38
		ARG 149	pi-H	4.26

Among the tested compounds, leonoside B (−6.54 kcal/mol) demonstrated the most favorable binding affinity, indicating a strong interaction with the active site of the enzyme. Leonoside B established both hydrogen-bond and π–H interactions with ARG149, suggesting a stable ligand–receptor complex (Figure 7). Such interactions may interfere with the normal function of microbial proteins, thereby inhibiting bacterial growth or metabolic processes.

**FIGURE 7:**
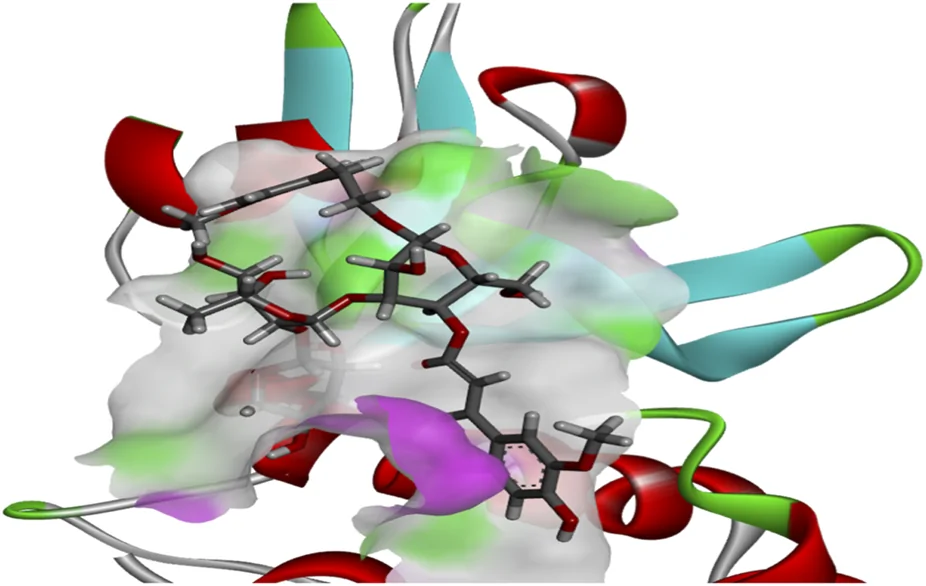
Docking of leonoside B and 1KZN protein.

Other compounds, including flavonoid derivatives and phenolic acids showed moderate binding energies, which may contribute collectively to the antimicrobial activity of the plant extracts *CL1* and *CL2*. The presence of multiple hydroxyl groups in these compounds facilitates the formation of hydrogen bonds with amino acid residues within the catalytic pocket of the target protein. These results correlate with the *in vitro* antimicrobial assay, where the phenolic-rich extracts of *C. ladanifer* demonstrated inhibitory effects against several microbial strains. Similar observations have been reported for other plant-derived flavonoids, which are known to inhibit microbial enzymes and disrupt bacterial cell membranes.

When compared with standard antimicrobial drugs such as ampicillin or ciprofloxacin, the binding affinities of the natural compounds are generally slightly lower; however, the capacity of these phytochemicals to interact with multiple residues within the active site suggests a potential synergistic effect. Such multi-target interactions are often associated with reduced microbial resistance and enhanced therapeutic potential.

#### 3.9.3 Antihyperglycemic activity

Docking analysis against the antihyperglycemic target protein (3TOP) revealed strong interactions between several *C. ladanifer* phytochemicals and the active site of the enzyme involved in carbohydrate metabolism. Binding energies ranged from −5.4 to −9.54 kcal/mol, indicating significant ligand–protein interactions (Table 11).

**TABLE 11:** Docking score and interaction between the compounds and 3TOP protein.

Compound	Binding energy (kcal/mol)	Receptor	Interaction	Distance
Caffeic acid	−5.4	ASP 1526	H-donor	3.49
		ASP 1279	H-donor	3.44
		ASP 1279	H-donor	3.03
		ASP 1157	H-donor	3.41
Flavanone 7-O-glucoside	−7.74	ASP 1279	H-donor	2.69
		ASP 1526	H-donor	2.59
		HIS 1584	H-acceptor	2.95
Punicalin isomer 1	−5.55	ASP 1370	H-donor	3.26
		ASP 1526	H-donor	3.16
Valoneic acid dilactone	−7.81	ASP 1157	H-donor	3.2
		ASP 1279	H-donor	2.73
		ARG 1510	H-acceptor	2.58
		LYS 1460	H-acceptor	3.46
Kaempferol	−6.14	ASP 1157	H-donor	3.4
		ASP 1279	H-donor	3.46
		MET 1421	pi-H	4.16
Quercetin 3,4′-diglucoside	−9.54	ASP 1157	H-donor	2.68
		ASP 1526	H-donor	2.62
		ASP 1279	H-donor	2.72
		HIS 1584	H-acceptor	2.89
		TRP 1369	pi-H	3.72
Apigenin 7,4′-dimethyl ether	−6.44	MET 1421	pi-H	4.15
Resveratrol 4'-(6-galloylglucoside)	−8.71	ASP 1279	H-donor	2.75
		ARG 1510	H-acceptor	2.96
		LYS 1460	H-acceptor	2.8
Leonoside B	−9.22	ASP 1157	H-donor	3.22
		ASP 1279	H-donor	2.88
		GLN 1372	H-donor	2.92
		THR 1586	H-acceptor	3.29

Among the tested compounds, Quercetin 3,4′-diglucoside exhibited the highest binding affinity (−9.54 kcal/mol), suggesting a highly stable complex with the receptor. This compound established multiple hydrogen bonds with key residues such as ASP1157, ASP1526, and ASP1279, as well as π–H interactions with TRP1369. The presence of multiple hydroxyl groups in the quercetin structure likely enhances its ability to interact with catalytic residues within the enzyme active site.

Other compounds such as Valoneic acid dilactone (−7.81 kcal/mol) and Flavanone 7-O-glucoside (−7.74 kcal/mol) also showed strong binding affinities through multiple hydrogen-bond interactions with catalytic residues such as ASP1279 and ARG1510. These amino acids are known to play an essential role in enzyme activity and substrate recognition.

In addition to these compounds, Leonoside B demonstrated notable interactions with the active site residues (Figure 8), suggesting that this molecule may also contribute to the antihyperglycemic potential of *CL2*.

**FIGURE 8:**
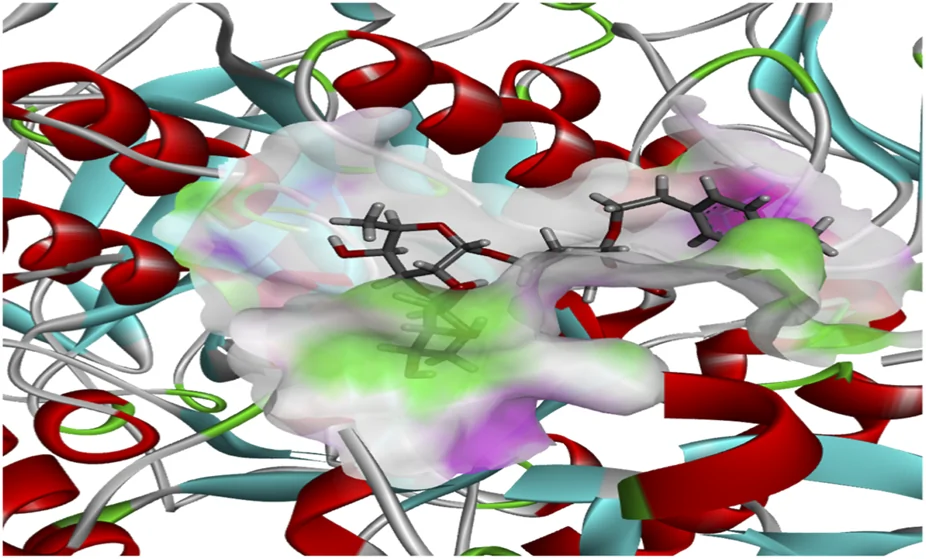
Docking of leonoside B and 3TOP protein.

The presence of several hydroxyl groups in its structure facilitates hydrogen-bond formation with amino acid residues located in the catalytic region of the protein, thereby enhancing the stability of the ligand–protein complex. Such interactions may inhibit enzymatic activity involved in carbohydrate hydrolysis, leading to reduced glucose release and improved glycemic control.

These findings are consistent with the *in vivo* antihyperglycemic activity observed for *CL2* during the OGTT, where the extract significantly reduced postprandial blood glucose levels. The docking results therefore support the hypothesis that phenolic compounds, particularly flavonoids and glycosylated derivatives such as quercetin and leonoside derivatives, contribute to the antidiabetic activity of the extract.

When compared with standard antidiabetic drugs such as acarbose or glibenclamide, several of the identified compounds showed comparable binding interactions within the catalytic pocket of the enzyme. This suggests that these natural compounds may act as potential inhibitors of carbohydrate-digesting enzymes, thereby slowing glucose absorption and contributing to the regulation of blood glucose levels.

## 4 Conclusion

The present study provides an integrated phytochemical and biological evaluation of *Cistus ladanifer* aerial part extracts. The results demonstrated that *CL2* was particularly enriched in total polyphenols, flavonoids, and condensed tannins and exhibited the strongest antioxidant activity in the TAC and DPPH assays, while showing comparable FRAP activity to *CL1*. The antimicrobial results further indicated that both extracts exhibited activity against several bacterial and fungal strains, with *CL2* showing greater activity against several of the tested microorganisms. HPLC/UV-ESI-MS profiling revealed a complex composition dominated by phenolic acids, flavonoids, and related phenolic constituents, providing a plausible chemical basis for the observed biological activities.

Based on its favorable chemical and biological profile, *CL2* was selected for further *in vivo* investigation. The acute toxicity study showed no observable toxicity at 2 g/kg, while the oral glucose tolerance test demonstrated a significant antihyperglycemic effect compared with the control group. Molecular docking further suggested that several identified phenolic compounds, particularly leonoside B and quercetin 3,4′-diglucoside, could interact with selected protein targets related to oxidative stress, antimicrobial activity, and glucose metabolism. However, these *in silico* findings should be considered as supportive evidence rather than definitive proof of the underlying mechanisms.

Overall, the findings highlight the biological potential of *C. ladanifer* aerial parts, particularly *CL2*, as a source of bioactive phenolic constituents. Further studies, including subacute and chronic toxicity investigations and mechanistic studies, are required to confirm its safety, clarify the mechanisms of action, and identify the compounds or combinations responsible for the observed activities.

## Data Availability

The original contributions presented in the study are included in the article/Supplementary Material, further inquiries can be directed to the corresponding author.

## References

[B1] Abdelfattah B. Mrabet A. Simou A. Khaddor M. (2024). Exploring the bioactive potential of *Cistus ladanifer* leaves from Northern Morocco (Tangier). Ecol. Eng. and Environ. Technol. 25, 316–330. 10.12912/27197050/187764

[B71] AFNOR. (1999). NF X31‐151: Qualité des sols — Mise en solution des éléments métalliques traces par attaque acide. Paris, France: Association Française de Normalisation (AFNOR)

[B2] Al-Khayri J. M. Sahana G. R. Nagella P. Joseph B. V. Alessa F. M. Al-Mssallem M. Q. (2022). Flavonoids as potential anti-inflammatory molecules: a review. Molecules 27 (9), 2901. 10.3390/molecules27092901 35566252 PMC9100260

[B3] Albus U. (2012). Guide for the Care and Use of Laboratory Animals. 8th edn.

[B4] Atanasov A. G. Zotchev S. B. Dirsch V. M. Supuran C. T. Banach M. Rollinger J. M. (2021). Natural products in drug discovery: advances and opportunities. Nat. Reviews Drug Discovery 20 (3), 200–216. 10.1038/s41573-020-00114-z PMC7841765 33510482

[B5] Balouiri M. Sadiki M. Ibnsouda S. K. (2016). Methods for *in vitro* evaluating antimicrobial activity: a review. J. Pharmaceutical Analysis 6 (2), 71–79. 10.1016/j.jpha.2015.11.005 29403965 PMC5762448

[B6] Basha N. J. Basavarajaiah S. M. Baskaran S. Kumar P. (2022). A comprehensive insight on the biological potential of embelin and its derivatives. Nat. Prod. Res. 36 (12), 3054–3068. 10.1080/14786419.2021.1955361 34304655

[B7] Benali T. Bouyahya A. Habbadi K. Zengin G. Khabbach A. Achbani E. H. (2020). Chemical composition and antibacterial activity of the essential oil and extracts of *Cistus ladaniferus* subsp. *ladanifer* and Mentha suaveolens against phytopathogenic bacteria and their ecofriendly management of phytopathogenic bacteria. Biocatal. Agric. Biotechnol. 28, 101696. 10.1016/j.bcab.2020.101696

[B8] Benali T. Laghmari M. Touhtouh J. Aanniz T. Lemhadri A. Daoudi M. D. (2024). Chemical composition and bioactivity of essential oils from *Cistus ladanifer* L., Pistacia lentiscus L., and Matricaria chamomilla L. Biochem. Syst. Ecol. 116, 104880. 10.1016/j.bse.2024.104880

[B9] Benamari O. Labhar A. Ahidar N. Salhi A. Elyoussfi A. Benyoub B. (2024). Investigation of the antioxidant and anti-inflammatory capacities of different extracts from *Cistus ladanifer* L. leaves in the ait ammart region (northern Morocco). Ecol. Eng. and Environ. Technol. 25, 178–184. 10.12912/27197050/179421

[B10] Bhattacharya M. Mandal S. (2025). Antibacterial activity of caffeic acid from plant sources: a review based on *in silico, in vitro* and *in vivo* approaches. Microbe 8, 100541. 10.1016/j.microb.2025.100541

[B11] Blasco F. J. D. Viciano-Tudela S. Parra L. Ahmad A. Chaloupková V. Bados R. (2024). Employment of MQ gas sensors for the classification of *Cistus ladanifer* essential oils. Microchem. J. 206, 111585. 10.1016/j.microc.2024.111585

[B12] Bouothmany K. Bourhia M. Aoussar N. Attaleb M. Salamatullah A. M. Nafidi H. A. (2022). Leaf extracts of *Cistus ladanifer* exhibit potent antioxidant and antiproliferative activities against liver, prostate and breast cancer cells. Appl. Sci. 12 (17), 8603. 10.3390/app12178603

[B13] Bouothmany K. Amrati F. E. Z. Chebaibi M. Bin Jardan Y. A. Nafidi H. A. Bourhia M. (2024). Hepatoprotective effects of a chemically-characterized extract from *Cistus ladanifer: in vivo* and *in silico* investigations. Nat. Product. Commun. 19 (2), 1934578X241228944. 10.1177/1934578x241228944

[B14] Bousta D. Farah A. El youbi-el Hamsas A. (2013). Phytochemical screening, antidepressant and immunomodulatory effects of aqueous extract of *Cistus ladanifer* L. from Morocco. Int. J. Phytopharm. 4, 12–17.

[B15] Chaloupková V. Mediavilla I. Bados R. Houdková M. Rondevaldová J. Esteban L. S. (2024). Annual variation in yield, chemical composition, antioxidant and antibacterial activity of rockrose (*Cistus ladanifer* L.) essential oils. Biocatal. Agric. Biotechnol. 60, 103279. 10.1016/j.bcab.2024.103279

[B16] Chang C. C. Yang M. H. Wen H. M. Chern J. C. (2002). Estimation of total flavonoid content in propolis by two complementary colorimetric methods. J. Food Drug Analysis 10 (3).

[B17] Comandini O. Contu M. Rinaldi A. C. (2006). An overview of cistus ectomycorrhizal fungi. Mycorrhiza 16 (6), 381–395. 10.1007/s00572-006-0047-8 16896800

[B18] Daglia M. (2012). Polyphenols as antimicrobial agents. Curr. Opinion Biotechnology 23 (2), 174–181. 10.1016/j.copbio.2011.08.007 21925860

[B19] Daoudi N. E. Bouhrim M. Ouassou H. Legssyer A. Mekhfi H. Ziyyat A. (2020). Inhibitory effect of roasted/unroasted Argania spinosa seeds oil on α-glucosidase, α-amylase and intestinal glucose absorption activities. South Afr. J. Bot. 135, 413–420. 10.1016/j.sajb.2020.09.020

[B60] da Silva A. P. G. (2021). Fighting coronaviruses with natural polyphenols. Biocatal. Agricultural Biotechnology 37, 102179. 10.1016/j.bcab.2021.102179 PMC8491928 34630764

[B20] Dong H. Song G. Wang Z. Wu X. Wang Q. Wang Y. H. (2025). Kaempferol as a multifaceted immunomodulator: implications for inflammation, autoimmunity, and cancer. Front. Immunol. 16, 1671519. 10.3389/fimmu.2025.1671519 PMC12408509 40918131

[B21] Drioiche A. Ailli A. Remok F. Saidi S. Gourich A. A. Asbabou A. (2023). Analysis of the chemical composition and evaluation of the antioxidant, antimicrobial, anticoagulant, and antidiabetic properties of Pistacia lentiscus from Boulemane as a natural nutraceutical preservative. Biomedicines 11 (9), 2372. 10.3390/biomedicines11092372 PMC10525226 37760813

[B30] El Hachlafi N. Kandsi F. Elbouzidi A. Lafdil F. Z. Nouioura G. Abdallah E. M. (2024). Extraction of bioactive compound-rich essential oil from *Cistus ladanifer* L. by microwave-assisted hydrodistillation: GC-MS characterization, *in vitro* pharmacological activities, and molecular docking. Separations 11 (7), 199. 10.3390/separations11070199

[B35] El Kabbaoui M. Chda A. El-Akhal J. Azdad O. Mejrhit N. Aarab L. (2017). Acute and sub-chronic toxicity studies of the aqueous extract from leaves of *cistus Ladaniferus* L. in mice and rats. J. Ethnopharmacology 209, 147–156. 10.1016/j.jep.2017.07.029 28750941

[B36] El Karkouri J. Sbai H. El Aggadi S. Bouyahya A. Zengin G. Harhar H. (2024). Phytochemical analysis, antioxidant, toxicity, and antihyperglycemic effects of *Cistus Ladaniferus* L., extracts. Microchem. J. 207, 111766. 10.1016/j.microc.2024.111766

[B22] Farhadi F. Khameneh B. Iranshahi M. Iranshahy M. (2019). Antibacterial activity of flavonoids and their structure–activity relationship: an update review. Phytotherapy Res. 33 (1), 13–40. 10.1002/ptr.6208 30346068

[B23] Ferreira S. Santos J. Duarte A. Duarte A. P. Queiroz J. A. Domingues F. C. (2012). Screening of antimicrobial activity of *Cistus ladanifer* and Arbutus unedo extracts. Nat. Product Research 26 (16), 1558–1560. 10.1080/14786419.2011.569504 22077559

[B24] Fernández-Arroyo S. Barrajón-Catalán E. Micol V. Segura-Carretero A. Fernández-Gutiérrez A. (2010). High-performance liquid chromatography with diode array detection coupled to electrospray time-of-flight and ion-trap tandem mass spectrometry to identify phenolic compounds from a *Cistus ladanifer* aqueous extract. Phytochem. Analysis PCA 21 (4), 307–313. 10.1002/pca.1200 20039337

[B25] Filali M. Lahyaoui M. Bahsis L. Rodi Y. K. El Hadrami E. M. (2024). Novel triazole-pyrazine as potential antibacterial agents: synthesis, characterization, antibacterial activity, drug-likeness properties and molecular docking studies. Moroc. J. Chem. 12 (3), 1367–1379 . 10.48317/IMIST.PRSM/morjchem-v12i3.46044

[B26] Frazão D. F. Raimundo J. R. Domingues J. L. Quintela-Sabarís C. Gonçalves J. C. Delgado F. (2018). *Cistus ladanifer* (cistaceae): a natural resource in Mediterranean-type ecosystems. Planta 247 (2), 289–300. 10.1007/s00425-017-2825-2 29218421

[B27] Frazão D. F. Martins-Gomes C. Steck J. L. Keller J. Delgado F. Gonçalves J. C. (2022). Labdanum resin from *Cistus ladanifer* L.: a natural and sustainable ingredient for skin care cosmetics with relevant cosmeceutical bioactivities. Plants 11 (11), 1477. 10.3390/plants11111477 35684251 PMC9183103

[B28] Frazão D. F. Martins-Gomes C. Díaz T. S. Delgado F. Gonçalves J. C. Silva A. M. (2024). Labdanum resin from *Cistus ladanifer* L. as a source of compounds with anti-diabetic, neuroprotective and anti-proliferative activity. Molecules 29 (10), 2222. 10.3390/molecules29102222 PMC11124373 38792084

[B29] Gago C. Bakchiche B. Djekhioua T. da Graça Miguel M. (2025). *Cistus ladanifer* L. essential oils, volatiles, by-products, and their biological properties. Molecules 30 (22), 4425. 10.3390/molecules30224425 PMC12655565 41302483

[B31] Hadi N. Drioiche A. Bouchra E. M. Baammi S. Abdelaziz Shahat A. Tagnaout I. (2024). Phytochemical analysis and evaluation of antioxidant and antimicrobial properties of essential oils and seed extracts of Anethum graveolens from Southern Morocco: *in vitro* and *in silico* approach for a natural alternative to synthetic preservatives. Pharmaceuticals 17 (7), 862. 10.3390/ph17070862 PMC11280095 39065713

[B32] Hayani M. Bencheikh N. Ailli A. Bouhrim M. Elbouzidi A. Ouassou H. (2022). Quality control, phytochemical profile, and antibacterial effect of Origanum compactum Benth. essential oil from Morocco. Int. J. Plant Biol. 13 (4), 546–560. 10.3390/ijpb13040044

[B33] Hussain A. (2025). Chronic hyperglycemia and cardiovascular dysfunction: an in-depth exploration of metabolic and cellular pathways in type 2 diabetes mellitus. Cardiovasc. Diabetology–Endocrinology Rep. 11 (1), 39. 10.1186/s40842-025-00247-3 PMC12699840 41382274

[B34] Joshi A. Bhobe M. Sattarkar A. (2013). Phytochemical investigation of the roots of Grewia microcos Linn. J. Chem. Pharm. Res. 5 (7), 80–87.

[B37] Köse M. D. Tekin B. N. Bayraktar O. Duman E. T. Başpınar Y. (2017). Antioxidant and antimicrobial properties of *Cistus ladanifer* . Int. J. Second. Metabolite 4 (3, Special Issue 2), 434–444. 10.21448/ijsm.376834

[B38] Lahyaoui M. Filali M. Benamar K. Sghyar R. Fikri-Benbrahim K. Haoudi A. (2024a). Development of novel antibiotics derived from pyridazine: synthesis, spectroscopic characterization, *in vitro* antimicrobial activity and molecular docking studies. Results Chem. 10, 101699. 10.1016/j.rechem.2024.101699

[B39] Lahyaoui M. Sghyar R. Benamar K. Filali M. Fikri-Benbrahim K. Kartah B. E. (2024b). Novel 1, 2, 3-triazolic compounds derived from cytosine: synthesis, spectroscopic characterization, *in vitro* antimicrobial activity and molecular docking studies. Results Chem. 7 101410, 10.1016/j.rechem.2024.101410

[B40] Li Y. Yao J. Han C. Yang J. Chaudhry M. T. Wang S. (2016). Quercetin, inflammation and immunity. Nutrients 8 (3), 167. 10.3390/nu8030167 26999194 PMC4808895

[B41] Liang H. Huang Q. Zou L. Wei P. Lu J. Zhang Y. (2023). Methyl gallate: review of pharmacological activity. Pharmacol. Res. 194, 106849. 10.1016/j.phrs.2023.106849 37429335

[B42] Lin C. C. Hsu Y. F. Lin T. C. Hsu H. Y. (2001). Antioxidant and hepatoprotective effects of punicalagin and punicalin on acetaminophen-induced liver damage in rats. Phytotherapy Res. Int. J. Devoted Pharmacol. Toxicol. Eval. Nat. Prod. Deriv. 15 (3), 206–212. 10.1002/ptr.816 11351354

[B43] Medina-Franco J. L. (2021). Computational approaches for the discovery and development of pharmacologically active natural products. Biomolecules 11 (5), 630. 10.3390/biom11050630 PMC8146100 33922650

[B44] Moumen B. E. Bouzoubaa A. Drioiche A. Eddahmouny M. Al Kamaly O. Shahat A. A. (2025). Unveiling the chemical composition, antioxidant, and antimicrobial potentials of Foeniculum vulgare mill: a combined *in vitro* and *in silico* approach. Int. J. Mol. Sci. 26 (10), 4499. 10.3390/ijms26104499 PMC12111592 40429645

[B45] Muscolo A. Mariateresa O. Giulio T. Mariateresa R. (2024). Oxidative stress: the role of antioxidant phytochemicals in the prevention and treatment of diseases. Int. Journal Molecular Sciences 25 (6), 3264. 10.3390/ijms25063264 38542238 PMC10970659

[B46] National Committee for Clinical Laboratory Standards Barry A. L. (1999). Methods for determining bactericidal activity of antimicrobial agents: approved guideline (Vol. 19, No. 18). Wayne, PA: National Committee for Clinical Laboratory Standards.

[B47] NFV03-402 (1985). Spices and Herbs—Determination of Water Content—Training Method. Paris, France: AFNOR.

[B48] NFV05-113 (1972). Fruits, Vegetables and Derived Products—Mineralization of Organic Matter—Incineration Method. Paris, France: AFNOR.

[B49] OECD (2002). “Test No. 423: Acute Oral Toxicity ‐ Acute Toxic Class Method,” in OECD Guidelines for the Testing of Chemicals, Section 4. Paris: OECD Publishing. 10.1787/9789264071001-en

[B50] Nortjie E. Basitere M. Moyo D. Nyamukamba P. (2022). Extraction methods, quantitative and qualitative phytochemical screening of medicinal plants for antimicrobial textiles: a review. Plants 11 (15), 2011. 10.3390/plants11152011 PMC9370299 35956489

[B51] Pandey A. Tripathi S. (2014). Concept of standardization, extraction and pre phytochemical screening strategies for herbal drug. J. Pharmacogn. Phytochemistry 2 (5).

[B52] Papaefthimiou D. Papanikolaou A. Falara V. Givanoudi S. Kostas S. Kanellis A. K. (2014). Genus cistus: a model for exploring labdane-type diterpenes' biosynthesis and a natural source of high value products with biological, aromatic, and pharmacological properties. Front. Chemistry 2, 35. 10.3389/fchem.2014.00035 24967222 PMC4052220

[B53] Prasannabalaji N. Muralitharan G. Sivanandan R. N. Kumaran S. Pugazhvendan S. R. (2012). Antibacterial activities of some Indian traditional plant extracts. Asian Pac. J. Trop. Dis. 2, S291–S295. 10.1016/s2222-1808(12)60168-6

[B54] Remok F. Saidi S. Gourich A. A. Zibouh K. Maouloua M. Makhoukhi F. E. (2023). Phenolic content, antioxidant, antibacterial, antihyperglycemic, and α-amylase inhibitory activities of aqueous extract of Salvia lavandulifolia vahl. Pharmaceuticals 16 (3), 395. 10.3390/ph16030395 36986494 PMC10051605

[B55] Saidi S. Remok F. Handaq N. Drioiche A. Gourich A. A. Menyiy N. E. (2023). Phytochemical profile, antioxidant, antimicrobial, and antidiabetic activities of ajuga iva (L.). Life 13 (5), 1165. 10.3390/life13051165 37240812 PMC10222250

[B56] Saleem A. Akhtar M. F. Sharif A. Akhtar B. Siddique R. Ashraf G. M. (2022). Anticancer, cardio-protective and anti-inflammatory potential of natural-sources-derived phenolic acids. Molecules 27 (21), 7286. 10.3390/molecules27217286 36364110 PMC9656250

[B57] Sghyar R. Lahyaoui M. Aflak N. Moussaoui O. Chda A. Bencheikh R. (2024). An investigation of novel series of 2-thioxo-1, 3-dithiol-carboxamides as potential antispasmodic agents: design, synthesis via coupling reactions, density functional theory calculations, and molecular docking. Molecules 29 (16), 3855. 10.3390/molecules29163855 PMC11356991 39202934

[B58] Sghyar R. Lahyaoui M. Rhazi Y. Aflak N. Moussaoui O. Chda A. (2025). Novel D-ribofuranosyl tetrazoles: synthesis, characterization, *in vitro* antimicrobial activity, and computational studies. ACS Omega 10 (2), 2116–2129. 10.1021/acsomega.4c08773 39866623 PMC11755160

[B59] Shaikh J. R. Patil M. (2020). Qualitative tests for preliminary phytochemical screening: an overview. Int. J. Chem. Stud 8 (2), 603–608. 10.22271/chemi.2020.v8.i2i.8834

[B61] Singleton V. L. Orthofer R. Lamuela-Raventós R. M. (1999). “[14] analysis of total phenols and other oxidation substrates and antioxidants by means of folin-ciocalteu reagent,” in Methods in Enzymology, 299. Academic Press, 152–178.

[B62] Skujins S. (1998). Handbook for ICP-AES (Varian-Vista). A short guide to vista series ICP-AES operation. Varian int. AG, Zug, version 1.0. Switz. Soc. Food Sci. Technol. 29, 221–227.

[B63] Smith-Hall C. Larsen H. O. Pouliot M. (2012). People, plants and health: a conceptual framework for assessing changes in medicinal plant consumption. J. Ethnobiology Ethnomedicine 8 (1), 43. 10.1186/1746-4269-8-43 23148504 PMC3549945

[B64] Sun B. Ricardo-da-Silva J. M. Spranger I. (1998). Critical factors of vanillin assay for catechins and proanthocyanidins. J. Agricultural Food Chemistry 46 (10), 4267–4274. 10.1021/jf980366j

[B65] Teixido A. L. Valladares F. (2015). Temperature-limited floral longevity in the large-flowered mediterranean shrub *Cistus ladanifer* (cistaceae). Int. J. Plant Sci. 176 (2), 131–140. 10.1086/679477

[B66] Tomou E. M. Lytra K. Rallis S. Tzakos A. G. Skaltsa H. (2022). An updated review of genus cistus L. since 2014: traditional uses, phytochemistry, and pharmacological properties. Phytochem. Rev. 21 (6), 2049–2087. 10.1007/s11101-022-09827-y

[B67] Trott O. Olson A. J. (2010). AutoDock vina: improving the speed and accuracy of docking with a new scoring function, efficient optimization, and multithreading. J. Comput. Chem. 31, 455–461. 10.1002/jcc.21334 19499576 PMC3041641

[B68] Unno T. Sugimoto A. Kakuda T. (2004). Xanthine oxidase inhibitors from the leaves of Lagerstroemia speciosa (L.) pers. J. Ethnopharmacology 93 (2-3), 391–395. 10.1016/j.jep.2004.04.012 15234783

[B69] Verma S. Singh A. Mishra A. (2013). Gallic acid: molecular rival of cancer. Environ. Toxicology Pharmacology 35 (3), 473–485. 10.1016/j.etap.2013.02.011 23501608

[B70] Wing Ying Cheung C. Gibbons N. Wayne Johnson D. Lawrence Nicol D. (2010). Silibinin--a promising new treatment for cancer. Anticancer Agents Med. Chem. 10 (3), 186–195. 10.2174/1871520611009030186 20015009

